# Monomers and short oligomers of human RAD52 promote single-strand annealing

**DOI:** 10.1073/pnas.2420771122

**Published:** 2025-04-04

**Authors:** Maria A. Kharlamova, Manish S. Kushwah, Tobias J. Jachowski, Sivaraman Subramaniam, Viktor Schiff, A. Francis Stewart, Philipp Kukura, Erik Schäffer

**Affiliations:** ^a^Cellular Nanoscience, Center for Plant Molecular Biology, University of Tübingen, Tübingen 72076, Germany; ^b^Department of Chemistry, Physical and Theoretical Chemistry Laboratory, University of Oxford, Oxford OX1 3QZ, United Kingdom; ^c^Genomics, Center for Molecular and Cellular Bioengineering, Biotechnology Center, Technische Universität Dresden, Dresden 01307, Germany; ^d^School of Biotechnology and Biomolecular Sciences, University of New South Wales, Sydney 2052, NSW, Australia; ^e^State Key Laboratory of Microbial Research, Shandong University, Qingdao 266237, China

**Keywords:** DNA repair, homologous recombination, single-strand annealing, mass photometry, RAD52

## Abstract

Genome maintenance and stability rely on the repair of DNA double-strand breaks. Breaks can be repaired via the single-strand-annealing pathway mediated by the protein RAD52. RAD52 oligomerizes to rings that are thought to promote annealing. However, rings have only been observed at micromolar concentrations at which annealing activity is impaired. Thus, it is unclear which oligomeric form is responsible for annealing. We combined single-molecule mass photometry with biochemical assays to determine the in vitro oligomeric states of human RAD52. We found that RAD52 was mostly monomeric at lower nanomolar concentrations. With increasing concentration, RAD52 oligomerized and formed rings with a variable stoichiometry from heptamers to tridecamers consistent with an oligomerization model of noncooperative assembly coupled with preferential cyclization. Under conditions where hardly any rings were present, RAD52 already promoted single-strand annealing in vitro. Our findings indicate that in vitro single-strand annealing can be mediated by monomers and short oligomers of RAD52. The oligomerization model suggests that ring formation is similar to a phase transition whereby rings are a reservoir to replenish the monomer and short oligomer pool. This pool has a nearly constant concentration which may be optimal for annealing and would be independent, for example, of the amount of DNA damage, protein upregulation, or the cell cycle.

## Significance

DNA repair is critical to maintaining genetic information. Accumulation of genomic errors can lead to serious diseases and cancer. Recently, RAD52, a repair protein involved in numerous pathways, has become a promising cancer drug target. However, how RAD52 performs its basic single-strand annealing function is unclear because its oligomeric state during repair is unknown. We present a concentration-dependent analysis of RAD52’s oligomeric state resolved by single-molecule mass photometry. Our results show that monomers and short oligomers are more abundant than ring structures at the net in vivo concentrations. We therefore propose that the more abundant species drive annealing. Our quantitative model suggests a biological function for the dilute phase following a condensation transition.

RAD52 is a single-strand annealing protein highly conserved in eukaryotes. Unlike RAD51/RecA, RAD52 is not an ATPase but still promotes annealing, first shown in vitro for yeast Rad52 (1). RAD52 is involved in many different repair pathways (2–13). It can either act independently or as a mediator for homologous recombination by recruiting RAD51 (9, 14). In mammals, this mediator activity is mostly done by BRCA2 (15). In BRCA2-deficient cancer cells, RAD52 compensates BRCA2’s function making it a target for cancer treatment (16–19).

**Fig. 1.**
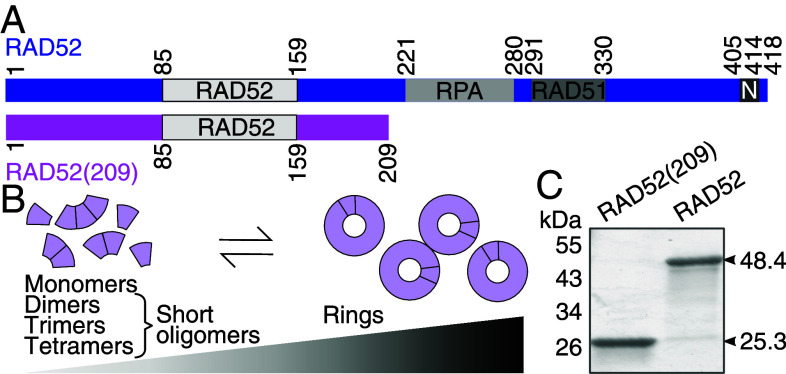
RAD52 domains and multimerization. (*A*) Domain schematic of full-length RAD52 and RAD52(209) including the self-association domain labeled RAD52, the RPA and RAD51 binding domains, and the nuclear localization domain N. (*B*) The oligomeric state depends on the protein concentration. Higher concentrations shift the equilibrium oligomeric state from short oligomers to rings and their clusters [$K_{d}\approx 14$ nM (52)]. (*C*) SDS-PAGE of purified RAD52 and RAD52(209) with indicated sequence mass.

RAD52 lacking its unstructured C-terminus is sufficient for single-strand annealing and oligomerization in vitro (Fig. 1) (6, 20–25). The N-terminal domain self-associates to form rings that can self-associate further to form larger ring clusters (26, 27). Early electron microscopy images show RAD52 as a heptameric ring with long protrusions attributed to the unstructured C-terminal domain (25, 27, 28). Further work showed that human RAD52 can also form undecameric rings (24, 25, 29) while yeast Rad52 forms decameric rings (30). Nonameric to undecameric rings are also formed by the C-terminally truncated RAD52 (20, 21, 23, 31).

In the inner groove of the ring, RAD52 specifically binds single-stranded DNA (ssDNA) (4, 23). This site binds four nucleotides compressed to a B-form DNA spacing (32) per RAD52 monomer (21, 23, 25, 33). A second site is located on the top of the ring that can also bind double-stranded DNA (dsDNA) (4, 34). This site promotes aggregation of RAD52 in the presence of ssDNA (23). RAD52 binds stably to ssDNA and in a diffusive manner to dsDNA (35).

Apart from the conspicuous ring structures, a range of shorter oligomers, larger particles, and various ring clusters have also been observed in vitro (25, 27, 36). RAD52 rings, their clusters, and larger assemblies can be disassembled by ssDNA, magnesium ions, or replication protein A (RPA) (29, 37). At low magnesium concentrations, RAD52 monomers preferentially localized in vitro at single-stranded 3’ ends such that they may protect the ends against exonucleases and serve as a nucleation site for the oligomerization of RAD52 from the 3’ end (29, 33, 38–40). In ssDNA–RPA–RAD52 complexes, rings were also not present (29).

With increasing RAD52 concentration, in vitro annealing rates and product yield first increase and then decrease again (6, 25, 41–43) with the maximum being in the lower nanomolar range corresponding to the estimated net in vivo concentrations (44–46). In vivo, RAD52 can form repair foci from liquid droplets at DNA breaks (16, 47–51) with the number of foci being less than the number of DNA breaks (47).

In vitro, RAD52 ring structures have only been observed at micromolar concentrations (20, 21, 23–25, 27–30), i.e., at conditions that impair annealing. Rings have been conjectured to be present at nanomolar concentrations, at which annealing assays are often performed, but there is no direct evidence for their presence or activity at these conditions. Recently, an apparent dissociation constant of 14 nM has been determined for RAD52’s self-association (52) (Fig. 1). To our knowledge, no study has so far directly visualized the nucleoprotein complex during annealing and the existence of ring structures has not been proven in vivo, yet (53). Thus, the oligomeric state of RAD52 that promotes annealing and how homology is detected is unknown.

Despite the evidence that RAD52 multimerization is concentration dependent, dynamic, and variable, current DNA annealing models are based on closed (4, 21, 23, 43, 54, 55) or open (25, 29) rings. In contrast, for the homologous, prokaryotic single-strand annealing protein Red*β*, which can also form rings and has the same, common protein fold for DNA binding as RAD52 and the single-strand annealing protein superfamily (53, 56, 57), a monomer-to-multimer model has been proposed (58–62).

To shed more light on how RAD52 anneals DNA, we determined the oligomeric state of full-length, human RAD52 in a minimal, in vitro annealing system in a concentration-dependent manner using mass photometry—a label-free, single-molecule technique that measures the molecular weight of individual proteins or complexes in their native state based on the interference contrast from light scattered back from surface-adsorbed molecules (63). We complemented these single-molecule experiments with modeling and electrophoretic mobility shift assays. We found that at lower nanomolar concentrations, at which annealing was already promoted, RAD52 was mostly present as monomers and short oligomers. Furthermore, the concentration dependence of oligomerization suggests that there is a critical concentration for ring formation in the lower nanomolar range below which hardly any rings exist. Our findings agree most with an annealing mechanism mediated by monomers and short RAD52 oligomers.

## Results

To determine RAD52’s annealing-active oligomeric state, we expressed full-length human RAD52 (RAD52, 48.4 kDa) and a construct truncated after amino acid 209 [RAD52(209), 25.3 kDa] in *Escherichia coli* and purified the proteins using affinity and size-exclusion chromatography (Fig. 1, *Materials and Methods*). At micromolar concentrations, native gels showed regular band patterns that we and others (64) have attributed to rings and their clusters (*SI Appendix*, Fig. S1 and section 1). Based on electrophoretic mobility shift assays, both proteins bound cooperatively to ssDNA (*SI Appendix*, Fig. S2, Tables S1 and S2, and section 1) and increased the annealing rate of complementary ssDNAs up to about 30× for RAD52 with a rate constant of 2.0±0.3 min^−1^ (≈0.03 s^−1^) for 100 nM RAD52 and 10 nM ssDNA compared to the hybridization control without protein (*SI Appendix*, Fig. S3 and Table S3).

### Micromolar RAD52 Concentrations Impair Annealing.

**Fig. 2.**
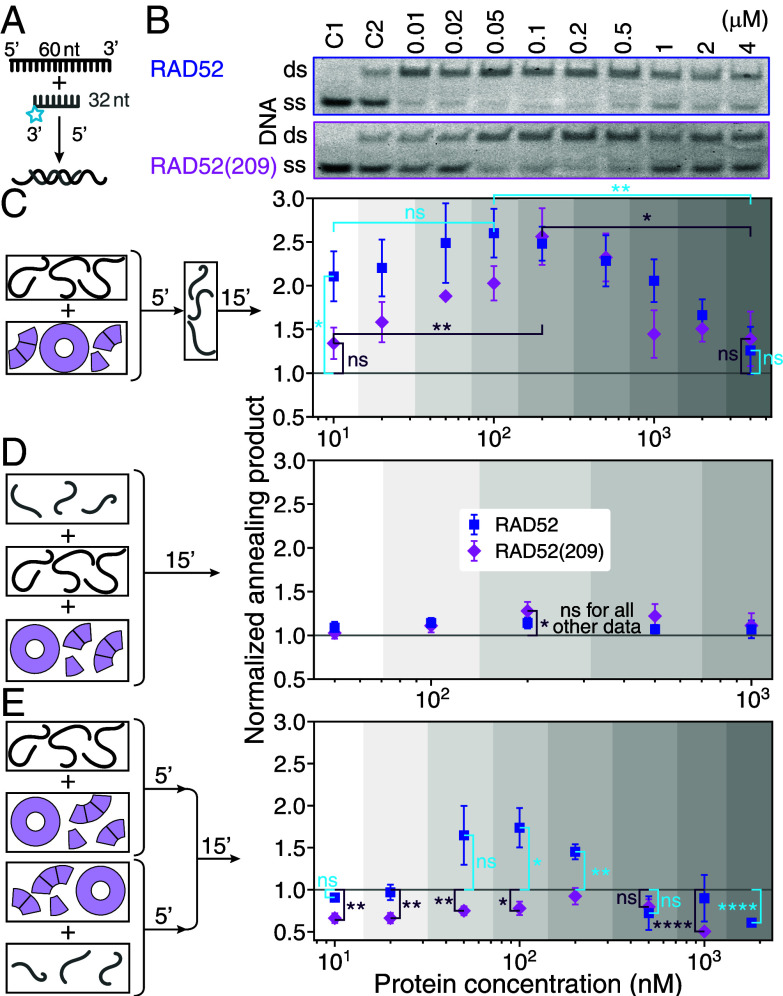
dsDNA product yield of the annealing reaction. (*A*) Hybridizing-reaction schematic of a 32-nt long, fluorescently labeled (gray with cyan star) and a 60-nt long, unlabeled (black) oligonucleotide without protein. (*B*) Annealing gels of electrophoretic mobility shift assays when one strand is incubated with the protein before mixing it with the other strand. Control C1: labeled oligonucleotide. C2: annealing control used for normalization, i.e. hybridizing reaction of both oligonucleotides without protein [gray horizontal lines in (*C*–*E*)]. Schematic and quantification of (*C*) the annealing reaction from (*B*) with one strand incubated with the protein before adding the other strand, (*D*) the annealing reaction with both strands and the protein mixed at the same time, and (*E*) the annealing reaction with both strands incubated separately with the protein before mixing. Incubation times in min are indicated on the arrows. All data points are mean values ± SEM (6 repeats) and oligonucleotide concentrations of both strands were 10 nM. Significance of differences is indicated (ns: not significant, *P ≤ 0.05, **P ≤ 0.01, and ****P ≤ 0.0001; see text).

There are two types of annealing models. In one, annealing occurs between two ssDNA–RAD52 complexes and in the other a ssDNA–RAD52 complex interacts with a bare or RPA-coated complementary ssDNA. To distinguish between these types and gain insight into the concentration dependence, we varied the order of incubation of RAD52 with its two complementary ssDNAs, a 32-nt, and a 60-nt long oligonucleotide (both 10 nM), as a function of protein concentration (Fig. 2, see *SI Appendix*, Fig. S4 and section 2 for other oligonucleotide pairs and concentrations, and *Materials and Methods*). When only one strand was incubated with the protein before adding the other strand, annealing product yield was increased up to about 2.5× for both proteins compared to the hybridizing reaction without protein (Fig. 2 *B* and *C*). At 10 nM RAD52, the yield was already increased by a factor 2.1±0.3 (mean ± SEM, *N* = 6; significantly different from the control: *P* = 0.012). Despite a 10× increase in protein concentration, the product yield enhancement at 100 nM of 2.6±0.3 was not significantly different (*t*-test: *P* = 0.24; an ANOVA test for all 10 to 100 nM data had no significance: *P* = 0.49; and there was also no significant correlation: *r* = 0.94 with *P* = 0.055). The yield at 4 μM of 1.3±0.3 was significantly decreased compared to the value at 100 nM (*P* = 0.0063) and not significantly different from the control level (*P* = 0.43). Correlated with product decrease, the amount of unreacted ssDNA increased (increase in ssDNA band intensity in Fig. 2*C* for ≥0.5 μM). At 200 nM RAD52(209), the maximum product yield of 2.6±0.3 was significantly increased compared to the value of 1.3±0.2 at 10 nM (*t* test: *P* = 0.008). The yield at 4 μM of 1.4±0.3 was significantly decreased compared to the value at 200 nM (*P* = 0.025). Both values at 10 nM and 4 μM were not significantly different from the control (*P* = 0.12 and 0.26, respectively). Thus, at micromolar concentrations for which we observed predominantly large protein complexes on native gels consistent with rings (*SI Appendix*, Fig. S1), annealing was not promoted by RAD52 and impaired compared to lower protein concentrations.

When both strands were directly mixed with the protein, annealing was not promoted except for RAD52(209) at 100 nM (1.3±0.1× with *P* = 0.042, Fig. 2*D*). When both strands were incubated with the proteins and subsequently mixed, annealing was also not promoted or even inhibited for most tested concentrations (Fig. 2*E*). Inhibition was particularly strong for RAD52(209) for which product formation was 2× reduced to 0.50±0.04 at 1 μM. Only for RAD52, annealing was significantly promoted at 100 nM and 200 nM that we attribute to RAD52’s C-terminal domain—the only difference between the two RAD52 constructs in these experiments. Together, the data of Fig. 2 implies that for efficient annealing, only one strand should be preincubated with the protein to form a ssDNA–RAD52 complex.

**Fig. 3.**
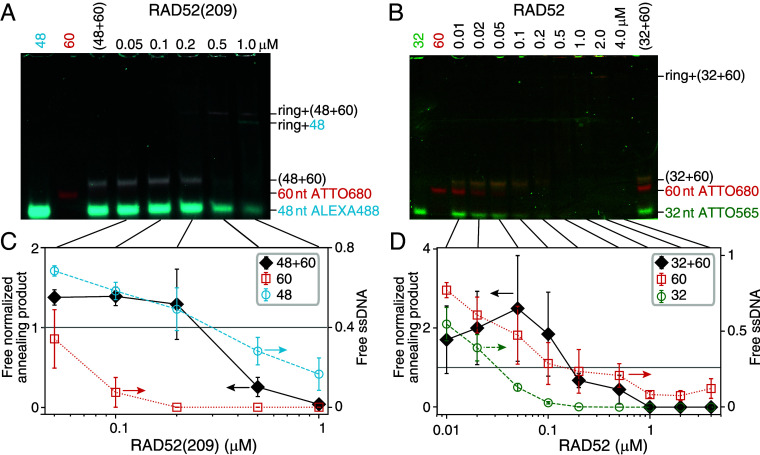
Annealing gels and product yield of fluorescently labeled oligonucleotides. (*A* and *B*) Native gels of complementary oligonucleotides with increasing concentrations of RAD52(209) and RAD52, respectively (the 60 nt-long strand was incubated with the protein before adding the other strand). The controls labeled with the oligonucleotide lengths were without protein. See *SI Appendix*, Fig. S5 for the separate color channels. Note that the gels are slightly distorted—the position of the short oligonucleotide band can be used as a reference. (*C* and *D*) Amount of annealing product (band intensity marked with the sum of the oligonucleotide lengths in (*A* and *B*), solid black diamonds) and unreacted ssDNA (open circles and red squares) normalized by control values plotted as a function of concentration (mean ± SEM, 6 repeats).

Previously, a substantial product increase was attributed to spontaneous annealing in the protease-containing stop buffer used for the above annealing assays (65) (*Materials and Methods*). To avoid effects due to deproteinization, we also ran native gels with fluorescent labels on both strands, one of which was preincubated with the protein (Fig. 3). In these assays, the protein can remain bound to the DNA. For RAD52(209) and RAD52 concentrations of ≤200 nM and ≤100 nM, respectively, the product yield was constant with values comparable to the 10 nM value in the deproteinization assay (RAD52: 1.94±0.15 and RAD52(209): 1.35±0.03 both significantly different from the control with *P* = 0.0001 and *P* = 0.007, respectively, Fig. 2*C*) but without the increase for RAD52(209). At higher concentrations, we observed a decline in product yield (consistent with Fig. 2) concurrent with the appearance of higher bands that we attribute to DNA-bound rings. Some of these bands contained both strands (*SI Appendix*, Fig. S5). Whether the two strands were also annealed or bound separately on different or the same ring is unclear. For RAD52(209), there was also an additional lower band containing only one strand (the shorter one, cyan in Fig. 3*A*). At ≥1 μM nearly all DNA was shifted to the higher bands or gel pockets implying that it was bound to rings, their clusters, or larger particles. Since the amount of annealing product declined at these concentrations in both annealing assays, these findings suggest that neither ring- nor larger-particle-bound DNA is beneficial for annealing.

### RAD52 Rings Are Variable in Protomer Number.

**Fig. 4.**
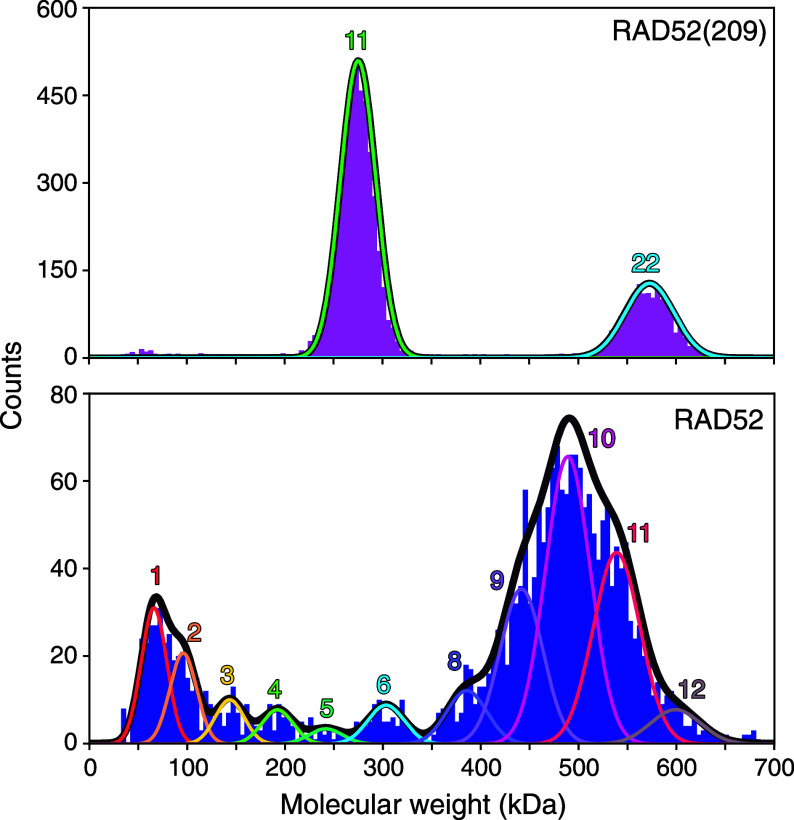
Mass-photometry molecular-weight histograms of RAD52(209) (*Top*) and RAD52 (*Bottom*) at 200 nM. Colored lines are fits of multiple Gaussians with numbers of protomers in the oligomers indicated and the envelope drawn in gray (see text for details). Each dataset corresponds to a single 60-s-long reading.

To measure the oligomeric state of individual complexes and their abundance under native conditions, we used mass photometry. We diluted the protein stock concentration to 1 μM and subsequently to the desired concentration. Samples were measured directly after the second dilution (*Materials and Methods*). At a concentration of 200 nM, the mass histogram of RAD52(209) had two peaks that were fitted by a sum of two Gaussians (Fig. 4, *Top*, see *Materials and Methods* for details on peak quantification and *SI Appendix*, Figs. S6 and S7 for buffer-control measurements). RAD52(209) formed undecameric rings with a molecular weight of 279.6 ± 1.3 kDa (mean ± SEM, *N* = 27 measurements) and clusters of two undecamers. The measured ring molecular weight is in excellent agreement with the calculated one of 278.3 kDa. The undecamer peak’s SD was 15.6 ± 0.5 kDa (mean ± SEM, *N* = 37, *SI Appendix*, Fig. S8), even smaller than those of proteins used for calibration (*SI Appendix*, Fig. S8). While the SD of the calibration peaks increased with molecular weight as expected (66, 67) (*SI Appendix*, Fig. S8), the small undecamer-peak SD indicates that the peak width is due to the measurement precision (66, 67) and does not arise from a variation in stoichiometry.

Interestingly, the mass histogram of RAD52 had peaks corresponding to monomers, short oligomers, and rings (Fig. 4, *Bottom*). The RAD52 ring SD when fitted to a single Gaussian was much larger than the expected one for a single oligomeric species (*SI Appendix*, Fig. S8). We attribute the large peak width to a convolution of multiple peaks consistent with the stoichiometry varying continuously between 8 to 12 protomers, with decamers having the highest abundance.

**Fig. 5.**
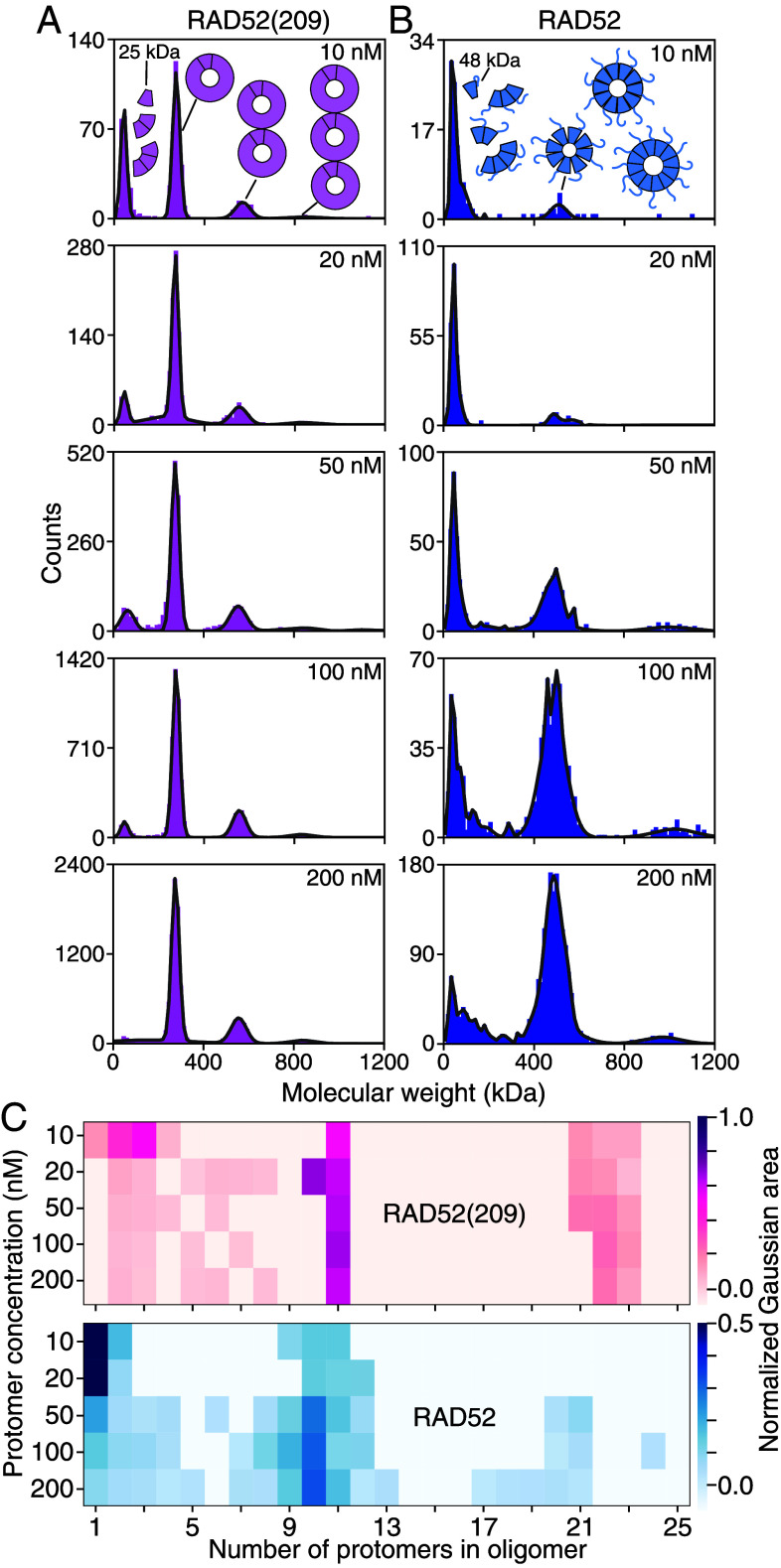
Mass-photometry molecular-weight histograms of (*A*) RAD52(209) and (*B*) RAD52 for increasing concentrations indicated in the panels. Lines are fits of multiple Gaussians. Each dataset is a cumulative distribution from four measurements on different days. Schematics next to or Above peaks illustrate the peak’s composition of short oligomers or rings. (*C*) Normalized abundance of oligomers as a function of concentration. The color scales on the *Right* represent the normalized area under each oligomer in (*A* and *B*).

To determine how the abundance of the oligomeric species depended on concentration, we measured mass histograms of both proteins as a function of protein concentration down to 10 nM (Fig. 5 and *SI Appendix*, Figs. S9 and S10 for recordings on different days). With lower concentrations, the abundance of monomers and short oligomers increased for both proteins indicated by higher peaks at lower molecular weights. The fidelity of detecting a RAD52(209) monomer was poor, however, because its molecular weight is below the 40 kDa-lower-mass-detection limit of the mass photometry instrument we used. On the increased molecular weight scale of Fig. 5, clusters of rings for both proteins are visible (at about 550 kDa and 800 kDa for RAD52(209), and 1 MDa for RAD52). RAD52 had less ring clusters, i.e. peaks at two and three times the ring molecular weight were smaller compared to RAD52(209). For micromolar concentrations (≥1 μM), we observed ring clusters on native gels for both proteins (*SI Appendix*, Fig. S1 and section 1).

### At Low Nanomolar Concentrations, Monomers and Short RAD52 Oligomers Are More Abundant than Rings.

**Fig. 6.**
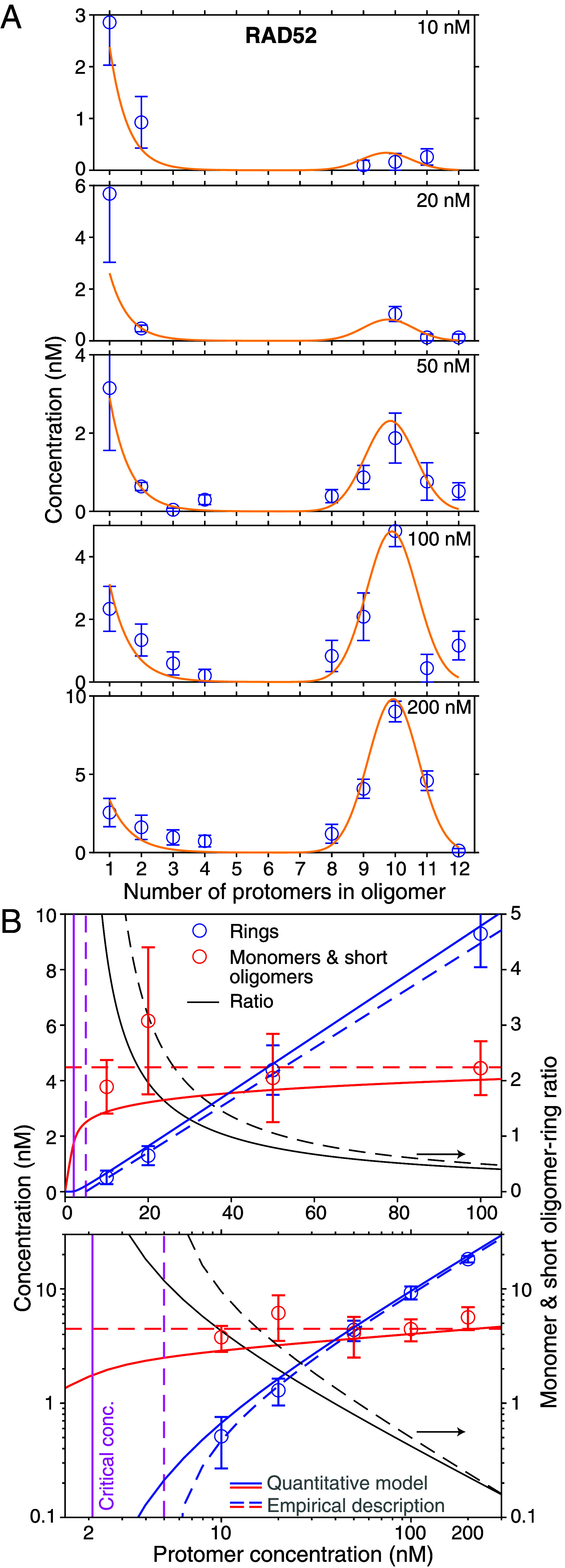
Quantitative RAD52 assembly model. (*A*) Global fit (yellow line) to oligomer concentrations (based on their counts in Fig. 5*B*, blue circles) as a function of RAD52 concentration. (*B*) Concentration of monomers and short RAD52 oligomers and rings (sums of 1 to 4 mer and 8 to 12mer concentrations, respectively, *Left* ordinate) and their ratio (black lines, *Right* ordinate) as a function of the protomer concentration (linear (*Top*) and logarithmic (*Bottom*) plots include zero and cover a larger concentration range, respectively). Lines are fits to the data (see text). Vertical magenta lines indicate the critical concentration.

Interestingly, the molecular-weight histograms (Fig. 5) had a multimodal distribution. If proteins self-associate to form linear aggregates or polymers, the length of such a linear polymer chain is expected to be short and exponentially distributed (see e.g. chapter 9 in ref. 68). Here, in contrast, there was a peak of oligomers with about 1 to 4 protomers—consistent with such an exponential—separated from the next peak containing oligomers of about 7 to 13 protomers (Fig. 5*C*). This separation implies that rings are thermodynamically more stable and associated with an extra energetic benefit compared to a linear chain with open ends (69). RAD52(209) formed more rings compared to RAD52 at low nanomolar concentrations. For RAD52, we detected a higher relative abundance of monomers and short oligomers compared to RAD52(209), although this comparison is not quantitative given the difference in detection fidelity for RAD52 vs. RAD52(209) monomers. Therefore, we restricted the following analysis to RAD52 as we could not robustly detect monomers of RAD52(209). For RAD52 at the lowest measured concentration of 10 nM, only few rings were present. When we determined the concentrations of the various RAD52 oligomers based on the counts, the analysis showed that there was an excess of monomers and short oligomers (monomers to tetramers summed together to 4±1 nM) over rings (0.5±0.2 nM, circles in Fig. 6). The ring abundance exceeded that of monomers and short oligomers at about 50 nM.

For an empirical description, we fitted lines to the data as a function of protomer concentration $c_{\text{protomer}}$ (dashed lines in Fig. 6*B*). The summed monomer and short oligomer concentration was on average, within error bars constant (4.5±0.4 nM, mean ± fit error, red dashed lines in Fig. 6). Interestingly, the ring concentration $c_{\text{ring}}$ increased in a linear fashion according to $c_{\text{ring}}=\left(c_{\text{protomer}}-c_{\text{crit}}\right)/n_{\text{avg}}$ with a significant offset corresponding to a critical concentration $c_{\text{crit}}$ for ring formation of 5.0 ± 0.7 nM (blue dashed lines in Fig. 6). The average number of protomers per ring was $n_{\text{avg}}$ of 10.6±0.2. Thus, according to this simple model, a minimum RAD52 concentration of about 5 nM is required for ring formation. In general, this critical concentration should be equal to the average monomer and short oligomer concentration above the critical concentration for multistranded filaments (68, 70, 71) (for example, for actin or microtubules) or filaments that close to a ring (69). Our data is consistent with this expected equality (5.0±0.7 nM vs. 4.5±0.4 nM, respectively).

To gain more quantitative insight into RAD52’s self-association, we fitted a model of noncooperative assembly coupled with preferential cyclization (69) to the data (solid lines in Fig. 6). The model is based on isodesmic growth (59, 70) characterized by a single dissociation constant *K*_*d*_ for a monomer associating with an oligomer containing *n* subunits. Due to an intrinsic curvature of the growing, semiflexible polymer, it can form a ring. When the ring closes, an extra bond is formed without the addition of another monomer and its associated, substantial entropy loss (69). Thus, ring closure lowers the free energy. A preferred curvature results in a ring with an optimal number of protomers *n*_*o*_ that minimizes elastic strain and the local free-energy during polymer growth. Yet, because the polymer is elastic and subjected to thermal bending motion, closed rings with a variable number of protomers can form with an elastic bending energy cost *E*_*b*_ of a Hookean spring. Thus, the formation of a closed ring from an open oligomer is modeled by an equilibrium constant *K*_*c*_ that depends on the size of the oligomer (69)

[1] $$K_{c}\left(n\right)=K_{c}^{o}e^{-E_{b}/\left(k_{\text{B}}T\right)}=K_{c}^{o}\exp\left[-\frac{1}{2}{\left(\frac{n-n_{o}}{\sigma_{\text{ring}}}\right)}^{2}\right],$$

where $\sigma_{\text{ring}}$ is the SD for the protomer number contained in a ring, $k_{\text{B}}$ the Boltzmann constant, and *T* the absolute temperature. The inverse variance $\sigma_{\text{ring}}^{-2}$ is proportional to an effective spring constant or bending stiffness. A global fit to the oligomer abundance including all data for 10 to 200 nM RAD52 (Fig. 6*A*) resulted in the dissociation constant $K_{d}=14\pm 2$ nM, the optimal ring protomer number $n_{o}=10.8\pm 0.1$ with a SD $\sigma_{\text{ring}}=0.79\pm 0.04$ and equilibrium constant $K_{c}^{o}=\left(1.9\pm 1.0\right)\cdot 10^{6}$ corresponding to a free energy gain for ring closure of $\Delta G_{\text{ring}}=k_{\text{B}}T\ln K_{c}^{o}\approx \left(14\pm 1\right)k_{\text{B}}T$. The *K*_*d*_ value is in excellent agreement with a recent report of 14±4 nM (52).

The quantitative model results in the same features as the empirical description (solid lines in Fig. 6*B*). The summed monomer and short oligomer concentration was nearly constant for a 10 to 200 nM protomer concentration with a modest increase of about a factor of 1.5. The ring concentration also had a critical concentration of ≈2 nM based on the maximum in curvature. Below this concentration, the monomer and short oligomer concentration exceeds the ring concentration more than 100-fold (152× at 2 nM). Thus, the most abundant RAD52 oligomeric species at low nanomolar, in vitro concentrations up to about 50 nM were monomers and short oligomers (Figs. 5 *B* and *C* and 6).

### ssDNA Binds All RAD52 Oligomeric Species and Aggregates RAD52 Rings.

**Fig. 7.**
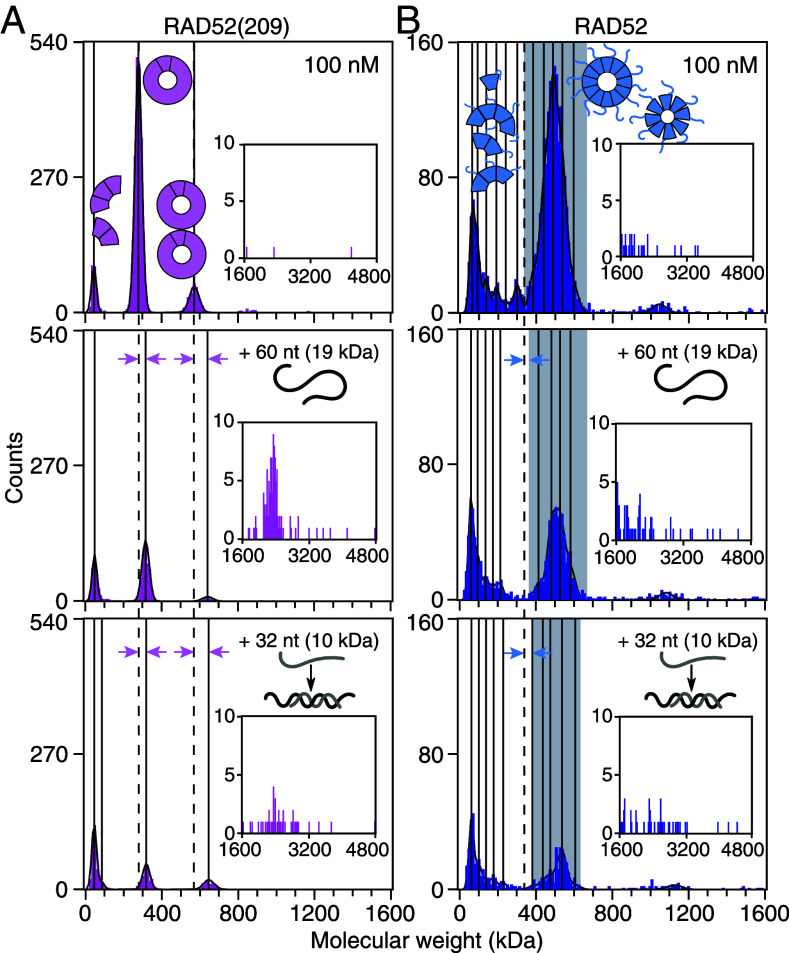
Annealing observed with mass photometry. (*A*) RAD52(209) and (*B*) RAD52 without DNA (both proteins 100 nM, *Top* row), in presence of ssDNA (10 nM, 60 nt, unlabeled, *Middle* row), and after the addition of the complementary strand (10 nM, 32 nt, unlabeled, *Bottom* row). Vertical solid lines indicate peak centers. Dashed lines mark RAD52(209) peak centers or the start of the RAD52 ring peak without added DNA. Molecular weight shifts upon DNA binding correspond to distance between arrowheads. Each dataset is a cumulative distribution from four measurements.

To detect on the single-molecule level ssDNA–RAD52 complexes, we monitored the annealing reaction via mass photometry (Fig. 7). After incubation of the first oligonucleotide (10 nM, 60 nt, 18.6 kDa, no fluorescent label) with the proteins (100 nM), the number of rings decreased and their molecular weight was shifted (*Middle* row Fig. 7). On these negatively charged, normal glass surfaces, the abundance and molecular weight of monomers and short oligomers did not change. On positively charged surfaces that enhance DNA binding (72), we also detected ssDNA-bound monomers and short RAD52 oligomers (*SI Appendix*, Fig. S11, Table S4, and section 3). However, these surfaces did not bind proteins without DNA well. On normal glass surfaces (Fig. 7), the well-defined RAD52(209) single- and double-ring peaks shifted by 42 ± 1 kDa and 74 ± 4 kDa, respectively, corresponding to about two and four bound ssDNA with a combined theoretical molecular weight of 37 kDa and 74 kDa, respectively (Fig. 7*A* and *SI Appendix*, Fig. S12). For shorter or longer strands, we found similar results (*SI Appendix*, Fig. S12). For RAD52, molecular-weight shifts were difficult to quantify due to the spectrum of ring structures (Fig. 7*B*). With an excess of ssDNA, rings were disassembled as previously suggested (29) (*SI Appendix*, Fig. S13 and section 4).

Apart from the expected ssDNA binding to monomers, short oligomers and rings (*SI Appendix*, Fig. S11 and Fig. 7), we observed an increased count of higher molecular-weight species (for example, a peak at ≈3 MDa for RAD52(209), *Insets* Fig. 7). In addition to these species, larger aggregates were visible in the raw images but were beyond the upper mass-calibration limit of around 6 to 7 MDa (*SI Appendix*, Fig. S14). We attribute the reduction in ring counts to all the large DNA–protein aggregates (>1.5 MDa) implying that they must have been largely composed of rings.

Addition of the complementary strand (10 nM, 32 nt, 9.8 kDa, no fluorescent label) reduced ring counts further but did not change the molecular-weight shift of ring peaks (*Bottom* row Fig. 7). Ring clusters were still present. RAD52(209) single-ring peaks shifted by 42 ± 2 kDa not significantly different from the shift when incubated with the first single strand (*SI Appendix*, Table S5). The dsDNA product’s molecular weight was below the detection limit. Taken together, we can conclude for the ssDNA–protein interaction that i) monomers and short oligomers can bind ssDNA (*SI Appendix*, Fig. S11), ii) two identical ssDNAs can bind a single ring (*Middle* row Fig. 7 and *SI Appendix*, Fig. S12), and iii) ssDNA can lead to RAD52(209) and RAD52 aggregate formation (*Insets* Fig. 7 and *SI Appendix*, Fig. S14).

## Discussion

Below we discuss the variation in RAD52 ring stoichiometry and how a microhomology search may accelerate homology detection. Such a search is based on a small recognition motif, with our biochemical data suggesting that a short RAD52 oligomer, rather than a ring, would be consistent with such a search mechanism. Next, based on our mass photometry data and quantitative modeling, we argue that RAD52 rings may act as a reservoir to buffer the pool of monomers and short oligomers, keeping their concentration constant and optimal for annealing under changing conditions during the cell cycle or upon DNA damage. We then outline an annealing mechanism based on cooperative binding, conformational proofreading for microhomology search, and DNA dissociation after successful homology detection. Finally, we speculate on how our findings fit into an in vivo context.

### RAD52 Forms Rings with a Variable Number of Protomers.

Consistent with the literature (20), we found that C-terminally truncated RAD52(209) mostly formed undecameric rings (Fig. 5*A*). For full-length RAD52, by contrast, we observed a spectrum of rings with a variable number of protomers from 7 to 13, with the decamer in highest abundance (Figs. 5*B* and 6*A*). When RAD52 was described to be a heptamer, the authors also noted the ring heterogeneity and broad width of its mass distribution (28), which is consistent with rings composed of 5 to 9 protomers. Other early measurements also indicated that RAD52 complexes may contain 4 to 13 protomers (27) or were “amorphous in shape” and variable in size with only occasional rings being visible (38). Hydroxyl radical treatment of ssDNA–RAD52 complexes revealed 4-nt binding per monomer and repeats up to 36 to 40 nt consistent with a maximum number of 9 to 10 protomers per ring (33). Cryo-EM data showed that RAD52 can form undecameric rings (24) or open rings with a smaller number of protomers ranging from 5 to 10 (25). Since experimental conditions were different, and relative abundances were not reported and may have been biased by using undecamer structures as initial models for classification or refinement, a direct comparison with mass photometry is difficult. Mass photometry cannot distinguish whether rings are open or closed. Our polymerization model implies that open and closed rings are in thermodynamic equilibrium with the equilibrium constant *K*_*c*_ given by Eq. **1**. Based on our *K*_*c*_ value, the open conformation is about a million-fold less likely. Our mass photometry data showed less frequent RAD52 ring formation with a more heterogeneous stoichiometry compared to RAD52(209) suggesting that the presence of the C-terminal region affects the self-association of RAD52 and could play an autoinhibitory role in ring formation. Taken together, our findings are consistent with previous reports and show that RAD52’s ring stoichiometry is variable. Thus, interpretations of data in terms of ring models that rely on a fixed stoichiometry may have to be revisited (for example, ref. 43).

### Annealing by ssDNA–RAD52 Complexes.

For RAD51, an eight-base-long microhomology unit is proposed to be an efficient recognition element that speeds up homology search (73, 74). By first identifying such a motif, the search space for neighboring homology is significantly reduced. Longer initial recognition motifs compromise reversibility and fidelity due to an enthalpic barrier (73). Shorter motifs significantly increase the search time because the search complexity increases exponentially as shorter sequences have many more exact matches in a genome (73). While the pathway, structure, and length of the recognition motif may be different for RAD52, the fundamental physical principles and boundary conditions for homology search are the same (74, 75). Since RAD52 binds ssDNA in units of four bases, a motif length of eight or twelve bases is plausible. In humans with a genome length of 3.2 Gb, the identification of ≈17 consecutive bases are required for unique homology (59, 73). Thus, at least a RAD52 tetramer binding 16 consecutive bases is necessary to identify homology with sufficient confidence after initial microhomology detection. Based on our data, we can narrow down possible homology detection and annealing mechanisms.

For RAD52, we measured an apparent *K*_*d*_ for ssDNA binding of about 100 nM with a Hill coefficient of H≈ 3 at 37 ^°^C (*SI Appendix*, Fig. S2 and Table S2). With an estimated diffusion limited on-rate on the order of $K_{\text{on}}=10^{8}$ M^−1^s^−1^ (76), the apparent off-rate $k_{\text{off}}=K_{d}K_{\text{on}}$ would be 10 s^−1^, which is much larger compared to our reaction rate for annealing of ≈0.03 s^−1^ (*SI Appendix*, Table S3). Thus, ssDNA binding and dissociation may not be rate-limiting at least for concentrations around or below the apparent *K*_*d*_. For higher concentrations, the oligomeric state may influence the off-rates significantly, as discussed below. Because on-rates can be much smaller due to conformational changes upon collision or larger due to a reduction of dimensionality by one-dimensional diffusion along DNA, these off-rates are only rough estimates. Nevertheless, these estimates provide boundary conditions and limits to annealing models. The Hill coefficient implies that RAD52 binds ssDNA cooperatively. Recent data also shows cooperative binding [reanalyzed data of supplementary figure 10*A* in ref. 52 with H=2.1±0.1 presumably at room temperature at which we also observed smaller *H*-values (*SI Appendix*, Table S2)]. The Hill coefficient is inconsistent with pure ring binding but would be consistent with a ssDNA–RAD52 complex consisting out of 2 to 3 consecutively bound protomers (*SI Appendix*, section 1 and Fig. S2). Such a small ssDNA–RAD52 complex could form the recognition motif for homology search.

For an identical ssDNA binding interface of RAD52 molecules, their binding energy would be equal. This equality would imply that the dimer *K*_*d*_ is the square of the one for the monomer. But due to entropic effects, number of available binding sites, and self-association for molecules binding cooperatively next to each other, the dimer *K*_*d*_ is expected to be larger compared to the squared value (59, 68, 69, 77, 78). For the RAD52 homologue Red*β*, each additional protomer lowered the complex dissociation constant by about 0.34 (59). Thus, we estimate that for an undecamer, the dissociation constant would be lowered by a factor $0.34^{10}\approx 10^{-5}$ compared to the monomer *K*_*d*_. Based on the structural and functional similarities in the RAD52 superfamily (53, 56, 57), a comparable decrease in the *K*_*d*_ for ssDNA–RAD52 binding as a function of oligomeric size could be expected. Thus, if our measured effective *K*_*d*_ of ≈100 nM is that of a dimeric or trimeric complex, we would expect for an undecamer a further reduction of at least $0.34^{8}\approx 2\cdot 10^{-4}$ to about $K_{d}^{\text{ring}}\approx 20$ pM corresponding to an off-rate of ≈0.002 s^−1^ using the above on-rate. This estimated small off-rate could explain the shift of ssDNA bands in native gels for increasing RAD52 concentrations consistent with ssDNA bound to rings (Fig. 3). Such a small off-rate could also compromise reversibility during homology search. Conversely, the monomer *K*_*d*_ should be a factor 3 to 9 larger ($0.34^{-1}$ to $0.34^{-2}$) than our measured apparent *K*_*d*_ which would significantly increase the off-rates for smaller oligomeric species. Unfortunately, the measurement of *K*_*d*_’s for individual oligomeric species is challenging but would provide significant insight into the homology search and annealing mechanism.

All ring-based annealing models assume that one strand is bound to a ring and the other strand either binds to the second DNA binding site on the ring or is bound to another ring (4, 21, 23, 43, 54, 55). For two strands of the same type bound to one ring (Fig. 7 and *SI Appendix*, Fig. S12), more complicated ring-based mechanisms would be required. If one strand is bound to the ring’s inner groove and annealing is mediated between two ring-bound ssDNA (23), steric hindrance between rings may keep the DNA strands too far apart for homology detection. Homology comparison beyond an 8-bp microhomology between two ring-bound DNAs would also be challenging because of the ring curvature. These considerations may explain why annealing was inhibited when both strands were incubated with the protein before mixing them at concentrations above 200 nM, for which rings were the most abundant species (Fig. 2*E*).

Consistently, reduced product yield correlated with high, ring-promoting concentrations [Figs. 2 and 3, and previous reports (6, 41–43)] suggesting that rings would impair annealing. If rings promote annealing, product yield should be proportional to the ring concentration (below saturation conditions). Between 10 to 100 nM RAD52, the ring concentration increased by 1,800% (Fig. 6*B*) that should result in a similar increase in product yield. However, product yield remained constant within error bars (Figs. 2*C* and 3) inconsistent with a ring-based annealing mechanism. Conversely, the measured summed monomer and short RAD52 oligomer concentration remained constant over this range (Fig. 6*B*). According to the quantitative model, the summed concentration increased by 40% which would suggest a similar increase in product. Such an increase would still be consistent with the data. These biochemical arguments suggest that monomers and short oligomers may drive product formation.

### RAD52 Rings May Act As a Reservoir.

Mass photometry enables the in vitro measurement of molecular weight and oligomerization state of native complexes at nanomolar concentrations on the order of the estimated net in vivo RAD52 concentration of 20 nM in yeast and 2 nM in humans (44–46). Considering the mammalian nuclear volume fraction to be at least 10% of the total cell volume, the nuclear RAD52 concentration would be less than 20 nM. Our data showed that at these concentrations, the RAD52 population predominantly consisted of monomers and short oligomers (Figs. 5*B* and 6).

Oligomerization and ring formation may be understood in terms of isodesmic growth (70) in combination with a cyclization model (69). Based on our data, the free energy gain for ring closure is about 14 $k_{\text{B}}T$ sufficient for ring stabilization. Thermal fluctuations may transiently open rings (25) that either reclose or dissociate into smaller subunits. Reversible ring formation is supported by the agreement of our model with the oligomer abundance distributions (Fig. 6) measured directly after samples were diluted 5 to 100×. If rings were very stable, i.e. would not fall apart after dilution, we would expect that the relative amount of monomers and short oligomers to rings should remain constant. However, we measured a more than 20 times increase in this ratio (see black lines in Fig. 6*B*) suggesting that samples quickly reached their equilibrium distributions in oligomer abundance and supporting the notion that ring formation is reversible.

Based on the quantitative oligomerization model, rings form and their concentration increases in a near linear fashion above a certain critical concentration while the monomer concentration remains nearly constant (69). This concentration dependence of monomers, short oligomers, and rings is consistent with our data (Fig. 6) and similar to that of the phage RAD52 homologue, Red*β* (59). A concentration dependence of this nature is similar to a phase transition, such as condensation (70, 71). In this analogy, rings would be the equivalent to the liquid phase and monomers and short oligomers would correspond to the gas phase with constant vapor pressure. The condensed liquid phase, the rings, would be a reservoir and the monomer/small oligomer concentration would remain nearly constant, independent of total RAD52 concentration once it is above the critical concentration. In this manner, the monomer and short oligomer concentration may be tuned to a constant, optimal concentration for annealing independent of the amount of DNA damage, cell growth, RAD52 upregulation, or the cell cycle. For example, during and after prometaphase, once the nuclear envelope breaks down, a large dilution of the RAD52 concentration up to an order of magnitude is expected as the protein would be distributed from the nucleus over the whole cell volume. Such a dilution should cause some rings to dissociate supported by our arguments on reversibility of ring formation—a prerequisite for a ring protein reservoir.

Our measured critical concentration of ≈2 to 5 nM for RAD52 ring formation is comparable to the estimated net in vivo concentration. Even though concentrations in the nucleus, phase-separated regions, or repair foci are higher, for mitotic DNA synthesis (7), one of RAD52’s key function, the net concentration should be the relevant one during mitosis. Whether in vivo, RAD52 is involved in single-strand annealing or performing one of its many other functions in phase-separated regions or foci, for example, acting as a mediator to recruit RAD51, is unclear. Such regions may also act as RAD52 reservoirs. The dilute phase of monomers and short oligomers may be the one responsible for annealing. Based on our data and theory, at lower nanomolar concentrations, RAD52 monomers and short oligomers dominate over rings. Based on abundance, these data suggest that the annealing-active form of RAD52 by itself may be monomers or short oligomers similar to its active form in ssDNA–RPA–RAD52 complexes (29) or its homologue Red*β* (59).

### A Dynamic Multistep, Monomer/Short-Oligomer-Mediated Annealing Mechanism.

**Fig. 8.**
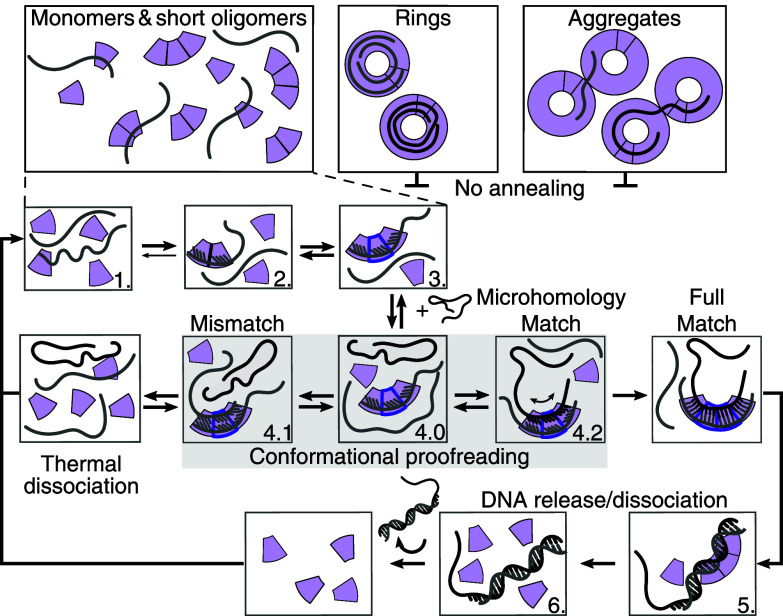
RAD52 single-strand annealing mechanism. Monomers and short RAD52 oligomers (purple annulus sectors) anneal complementary ssDNA strands (gray and black lines) in a multistep, catalytic mechanism. Rings or their aggregates inhibit annealing. See text for details.

Building on previous work, our data, and physical principles for homology search, we propose the following homology search and annealing model for RAD52 (Fig. 8). Due to functional and structural similarities within the RAD52 superfamily, we also refer to the Red*β* model that relies on monomer oligomerization on ssDNA (53, 59, 61, 62). Our RAD52 model is based on sequential, cooperative association of RAD52 monomers on ssDNA resulting in a short ssDNA–RAD52 complex that enables homology search with a complementary strand free of RAD52 via microhomology recognition. To explain annealing impairment at micromolar protein concentrations, we propose that rings and larger clusters or aggregates of rings stably bind ssDNA and inhibit annealing (Fig. 8, *Top*). Even though ring-bound ssDNA could act as a substrate for microhomology search and could contribute to annealing, we expect that reversibility is compromised due to the large microhomology length of at least 28 bp for the smallest rings consisting of 7 protomers (73). Rings would dynamically trap ssDNA due to their expected high affinity and low off-rate.

For microhomology search, monomers bind in a consecutive, cooperative manner forming a ssDNA–RAD52 complex consisting of 2 to 3 protomers (Fig. 8 Step 1 to 3). We expect that the DNA-interaction promotes RAD52 oligomerization in particular for concentrations around or below the self-association *K*_*d*_ of about 14 nM. The RAD52-related protein LiRecT required ssDNA for oligomerization (56). Without ssDNA, RAD52 monomers were the most abundant species for concentrations ≤50 nM (Fig. 6*A*). Without RAD52, complementary strands also hardly interacted and annealed with a time constant of ≈15 min (*SI Appendix*, Table S3). Once RAD52 and ssDNA interact, RAD52 oligomerizes on the ssDNA and promotes annealing accelerating annealing 30× to a time constant of about 30 s. We propose that ssDNA is bound at the inner site where ssDNA is compressed to a near B-form spacing with a stretched phosphate backbone every four nucleotides (23). In this manner, conformational proofreading (Fig. 8 Step 4.0) with a complementary strand allows for mismatch discrimination (59, 74, 75, 79, 80). Such a mechanism is supported by experiments with Red*β* that also compresses the extended base-pair spacing of ssDNA to almost that of B-form dsDNA but with a planar, unwound conformation upon annealing (57, 59). Similarly, for LiRecT, the average spacing is 3.8 Å with a stretched backbone every five nucleotides also in an unwound conformation (56). During conformational proofreading, only complementary strands bind for a sufficiently long microhomology search time. Without a match (Fig. 8 Step 4.1), the complex dissociates due to thermal fluctuations—an important step in homology detection in particular for a protein that is not an ATPase. If both strands are bound in a compressed form to RAD52, bases may not be accessible (23), conformational proofreading would be compromised, and the complex should also dissociate. This aspect may explain why preincubating both strands with protein does not promote annealing (Fig. 2*E*). In case of a microhomology match (Fig. 8 Step 4.2), at least one more RAD52 monomer should bind to the ssDNA–RAD52 complex to probe contiguous bases. With at least four protomers, the nucleoprotein complex is now large enough for unique homology identification (73, 74), yet not too large for dissociation in case that the sequence next to the probed microhomology region is not complementary. For complementary strands, the dsDNA product is released (Fig. 8 Step 5 and 6, consistent with the native gels for concentrations <200 nM, Fig. 3). Dissociation of the product implies a higher off-rate and *K*_*d*_ for the dsDNA–RAD52 interaction which has been shown to be of a diffusive nature (35). For Red*β*, a structural change upon nucleoprotein filament formation was found that led to a very stable filament (59). Whether there is a structural change for RAD52 and whether it could have the opposite effect of DNA dissociation compared to Red*β*’s filament stabilization is unclear. Dissociation might be driven by dsDNA relaxing to its straight, rod-like helical structure for lengths below its persistence length. Overall, the stepwise assembly of a nucleoprotein complex would enable harnessing both self-association and base-pairing energies for efficient, robust homology detection and release of a successfully annealed dsDNA product. In this sense, RAD52 may truly act as a catalyst without the need to hydrolyze ATP to promote annealing.

### Concluding Remarks.

While we could not directly visualize or detect the annealing process by RAD52, our data are most consistent with a monomer-driven model. While we cannot rule out annealing by rings, biochemical and reversibility arguments speak against ring models. The C-terminal domain of RAD52 may autoinhibit ring formation as it modulates the self-association of RAD52 resulting in more monomers and less rings of the full-length protein compared to the truncated one. According to our model, both factors would promote annealing which is supported by our data comparing the two constructs (Fig. 2*C*). In vivo, the C-terminal domain is key for interacting with RPA and RAD51. It may also interact with the complementary strand during annealing mediating an efficient scanning process for microhomology detection and/or the release of the dsDNA product (30). For successful annealing, we think that both DNA base pairing and RAD52 self-association are important. If the latter is disrupted, the protein would—according to our model—not promote annealing anymore, which is consistent with experiments using small-molecule inhibitors (18, 36). How oligomerization is affected by the crowded conditions inside cells, future experiments will have to tell. The same is true for how the ionic strength and the presence of divalent salts affect oligomerization and annealing. Binding affinities have been reported to increase in the presence of divalent cations but RAD52 “patch” sizes on ssDNA did not depend much on Mg^2+^ (35). Increased KCl concentrations led to RAD52 monomerization (81). Estimated physiological net RAD52 concentrations are in the lower nanomolar range. At these concentrations, we observed only few larger oligomers and rings in vitro. These may be necessary for localization to the nucleus (82). Apart from localization, rings may serve as a protein reservoir that is disassembled upon DNA damage or nuclear envelope break-down.

Since there are many competing biochemical reactions, most of which are not quantitatively characterized, yet, homology search, annealing, and inhibition reactions are still challenging to understand. To test our RAD52 short-oligomer-mediated model, further experiments are necessary that investigate the dynamics and visualize the process, for example, by simultaneous mass photometry and single-molecule fluorescent imaging, ideally also in the presence of RPA. The in vivo function of RPA is to protect ssDNA and prevent secondary structure formation of longer strands (65). In vitro, it is not essential for the annealing of short strands by RAD52. The RPA heterotrimer binds 30 nt and displacement of a single one may be sufficient for homology search. If RPA is displaced on one strand by a ssDNA–RAD52 complex that then interacts with a second RPA-coated strand, this situation may be analogous to our in vitro preincubation of only one strand with RAD52 (Fig. 2*C*). The complexity on the single-molecule level of the assembly of an RPA heterotrimer and its polymerization together with the oligomerization of RAD52 on ssDNA with subsequent annealing goes beyond the scope of the present work. We believe that our current findings using a minimal system contribute to a better understanding of the single-strand annealing mechanism by RAD52 and will help to develop drugs that efficiently target it for cancer treatments.

## Materials and Methods

### Single-Molecule Mass Photometry.

Mass photometry was performed on a commercial instrument OneMP (REFEYN Inc., Oxford, United Kingdom) using the regular field of view for the measurements and standard averaging parameters. Only for the RAD52 annealing measurements (Fig. 7), the medium field of view was used to increase RAD52 counts. The mass range of single, label-free proteins in their native state that can be detected quantitatively is 40 kDa to 5 MDa. Samples were diluted in Buffer 1 (20 mM Tris-HCl, 50 mM NaCl, pH 7.5). Purified Type 1 water (18.2 MΩ cm, Nanopure System MilliQ reference with Q-POD and Biopak filter) was used for all experiments. Buffers were filtered two times (0.22 μm pore size filter units, Merck) and degassed for 30 min. Glass slides (25 × 50 mm) were sonicated first in water, then in isopropanol, and again in water, each time 5 min at room temperature. Afterward slides were dried with filtered air. Cell culture gaskets were placed on top of the slides to contain the samples. The system was calibrated with NativeMark Unstained Protein Standard (Invitrogen, Cat. #LC0725, 1:400 dilution). Buffer 1 was measured on each day prior to experiments showing very low background counts (*SI Appendix*, Fig. S7). Acquired movies with binding events were processed in DiscoverMP (REFEYN Inc.). Generated HDF5 files were processed and fitted using a custom-written Python script. To determine the abundance of the individual oligomeric species, we fitted multiple Gaussian peaks to the mass histograms. Since peaks are overlapping, we used our knowledge of how the SD of the peaks depends on molecular weight. In this “deconvolution” approach, we used center values of the peaks corresponding to multiples of the monomer molecular weight and Gaussian peak SDs corresponding to the expected ones according to the linear dependence in *SI Appendix*, Fig. S8 as initial fit parameters. We allowed the centers, their SD, and their amplitude to vary for an optimal fit and limited the SD to a maximum value given by the linear dependence of the calibration-proteins’ SD (smaller values compared to the calibration proteins are possible, as we observed for the RAD52(209) undecamer, but not larger ones). Resulting fits represent envelopes of multi-Gaussian fits for each oligomeric state of the protein. Peaks with areas below 2% were excluded from the analysis. We varied the number of peaks and chose the optimal number based on the Akaike information criterion.

### Protein Purification.

The pET-15b vector harboring full-length human RAD52 and truncated RAD52(209) expression plasmids were gifts from Prof. Wataru Kagawa (Meisei University, Tokyo, Japan) and Prof. S. C. West (Cancer Research UK), respectively. Both constructs have a His-tag for purification. To overexpress proteins, the *E. coli* strain BL21(DE3) was cotransformed with the pET-15b vector containing the RAD52 genes and the pRARE vector, encoding low abundance tRNAs. For the purification, 6 and 4 L for RAD52 and RAD52(209), respectively, of a lysogeny broth culture supplemented with ampicillin and chloramphenicol were inoculated with fresh overnight culture and incubated at 37 ^°^C, and protein expression was induced at an optical density (A_600) of 0.8 with 0.5 mM isopropyl 1-thio-*β*-D-galactopyranoside (final concentration). Subsequently, the temperature was lowered to 24 ^°^C. Induction was carried out overnight. After induction, cells were harvested, resuspended in ≈80 ml of Buffer A (50 mM Tris-HCl, pH 7.8, 1.0 M KCl, 2 mM 2-mercaptoethanol, and 10% glycerol) containing 10 mM imidazole and a protease inhibitor cocktail (Merck KgaA, Germany), and lysed by sonication. All procedures after cell harvesting were performed at 4 ^°^C. The cell lysate was cleared of insoluble material by centrifugation at 40,000 g for 60 min. The supernatant was loaded onto a Buffer-A-equilibrated Histrap Ni-NTA fastflow (5 ml) column (Cytiva) used with an ÄKTAexplorer (Cytiva). After loading cell lysates, washing was carried out for 25 column volumes (125 ml) using Buffer A. Proteins were eluted with a 100-ml linear gradient of 50 to 500 mM imidazole in Buffer A. Fractions were collected from the peak and their purity was confirmed by 8% and 12% sodium dodecyl sulfate-polyacrylamide gel electrophoresis (SDS-PAGE) for RAD52 and RAD52(209), respectively. The pure fractions were loaded onto a Hiload 16/600 Superdex 200 pg column (Cytiva) after equilibration with RAD52 Buffer (50 mM KH_2_PO_4_, pH 8.0, 1 M KCl, 1 mM MgCl_2_, and 0.1 mM EDTA) and RAD52(209) Buffer (10 mM KH_2_PO_4_, pH 7.5, 10 mM NaCl, 1 mM dithiothreitol (DTT), and 0.1 mM EDTA) for RAD52 and RAD52(209), respectively. The fractions were collected from the eluted peak and controlled by an SDS-PAGE for RAD52 and RAD52(209), respectively. Pure fractions were concentrated to a final stock concentration of 50 μM (2.4 mg/ml) and 190 μM (4.8 mg/ml) for RAD52 and RAD52(209), respectively, using an Amicon Ultra 15 centrifugal filter (50 K MWCO and 30 K MWCO for RAD52 and RAD52(209), respectively) (Merck KgaA, Germany). Protein concentrations were determined using a NanoDrop 1000 (Thermo Fisher Scientific, Germany) with 20,587.5 M^−1^cm^−1^ and 40,380 M^−1^cm^−1^ as extinction coefficients for RAD52(209) and RAD52, respectively. The coefficients were calculated based on the amino acid composition of the proteins. We used 1.5 μL of freshly purified protein solution for a single measurement. We repeated measurements 3 to 4 times and averaged the values. The concentrated proteins were flash frozen in liquid nitrogen and stored at −80 ^°^C.

For all mass-photometry and gel-based experiments, we diluted the stock concentrations by the same procedure. Dilutions were done in two steps. First, we diluted the stock concentration to an intermediate concentration of 1 μM. Subsequently, we kept the protein on ice for 1 to 1.5 h during the experiments. The intermediate concentration was diluted further to the desired concentration and used immediately, typically within a few minutes after dilution. Each concentration was typically measured three times: directly, 2, and 4 min after dilution, with each mass photometry reading lasting 1 min. Relative mass histogram distributions hardly changed over this time, with a minor, expected reduction in the count rate. Since RAD52 is a sticky protein, all protein handling was done using low-binding pipette tips and tubes to minimize protein loss.

### ssDNA Binding Assays.

ssDNA binding was quantified by performing electrophoretic mobility shift assays with ssDNA of different lengths (*SI Appendix*, Table S1). Oligomer sequences were designed for minimal secondary structure formation and purchased from Sigma-Aldrich and Eurofins. Each oligonucleotide was fluorescently labeled at the 3’-end with ATTO565, ATTO680, or ALEXA488. RAD52(209) and RAD52 were diluted to desired concentrations in Buffer 2 (25 mM Tris-Acetate, 2 mM Mg(OAc)_2_, 1 mM DTT, pH 7.5). Proteins were incubated with 10 nM ssDNA either at 25 ^°^C for 10 min, or at 37 ^°^C for 5 min. Afterward, a gel loading dye (New England Biolabs, Cat. # B7024S) was added. Bands were resolved in precooled Tris-acetate-EDTA (TAE) buffer on a 10% TGX (Tris-Glycine eXtended)-Gel (BioRad). Gels were running at 4 ^°^C and 100 V (≈30 to 35 mA) until the lower front of the gel loading dye approached the bottom of the cassette. Gels were imaged on a Typhoon FLA 9000 (Cytiva). Band intensities were quantified in Fiji (83). All intensities were normalized to the control value (ssDNA, no protein added). These values were converted to percent and subtracted from 100%. Final values represent the amount of ssDNA bound to the protein as a function of protein concentration based on the decrease in intensity of bands running at the level of the control ssDNA without protein. Hill equations were fitted to the data weighted with error bars. Fitting constants are given in *SI Appendix*, Table S3.

### Terminated DNA Annealing Assays.

Annealing assays were performed on different pairs of ssDNA (*SI Appendix*, Table S1). Oligonucleotide concentrations were 10 nM. Protein concentrations were 100 nM. RAD52(209) and RAD52 were diluted to the desired concentrations in Buffer 2 and incubated with unlabeled ssDNA at 37 ^°^C for 5 min. Subsequently, complementary 3’-end-fluorescently labeled ssDNA was added and annealing proceeded at 25 ^°^C. To quantify the annealing rate, reactions were terminated after 1, 3, and 8 min with the STOP-Buffer (5 μM unlabeled ssDNA identical in sequence to the fluorescently labeled one, 10% SDS, 100 mM EDTA, and 10 units Proteinase K) in a 1:5 STOP-Buffer:reaction-mixture volume ratio and incubated 15 min at 30 ^°^C. Band intensities were quantified in Fiji and normalized to the control value (ssDNA at 0 min). These values were converted to percent and subtracted from 100%. Final values represent the amount of ssDNA that is annealed at different time points.

To determine the concentration of highest annealing activity, RAD52(209) and RAD52 were diluted in Buffer 2 to desired concentrations in a range from 10 nM to 10 μM. Some protein solutions were preincubated with a 60 nt-long, unlabeled oligonucleotide at 37 ^°^C for 5 min. Afterward a complementary 32 nt-long, 3’-end-ATTO565-labeled oligonucleotide was added. Final oligonucleotide concentrations were 10 nM. The annealing reaction proceeded at 25 ^°^C and was terminated after 15 min as described above. Subsequently, gel loading dye was added. Bands were resolved in precooled TAE buffer on a 10% TGX-Gel. Gels were running at 4 ^°^C and 100 V (≈30 to 35 mA) for 90 min and imaged using a Typhoon FLA 9000. Band intensities were quantified in Fiji and normalized to the control value (Column C2 in Fig. 2, dsDNA without protein). These values represent the amount of dsDNA product formed at different concentrations.

### Native DNA Annealing Assay.

To visualize intermediate species formed during the annealing reaction, proteins were preincubated at 37 ^°^C for 5 min in Buffer 2 with a 60 nt 3’-ATTO680 labeled oligonucleotide. Subsequently, a complementary, fluorescently labeled oligonucleotide was added (48-ATTO488 or 32-ATTO565). DNA concentrations were 10 nM. Protein concentrations were in the range from 50 nM to 2 μM. Annealing proceeded at 25 ^°^C for 15 min. Reactions were not terminated and immediately loaded on a gel with a nondenaturing gel loading dye (Thermo Fischer, Cat. #R0611). Bands were resolved in precooled TAE buffer on a 10% TGX-gel. Gels were running at 4 ^°^C and 100 V (≈30 to 35 mA) until the lower front of the gel loading dye approached the bottom of the cassette. As previously described, gels were imaged with a Typhoon FLA 9000 scanner with two excitation wavelengths. Band intensities of both ssDNA and dsDNA were quantified in Fiji. To quantify the amount of unreacted ssDNA, acquired intensities of the ssDNA were normalized to the corresponding control values of ssDNA (48 nt and 60 nt, respectively). To quantify the amount of non-protein-bound dsDNA product, intensities of bands running at the level of the control were normalized to the control value (no protein added). Final values were plotted as functions of protein concentrations.

### Blue Native Gels.

The native oligomeric state of the proteins at micromolar concentrations was visualized in blue native gels. Proteins were diluted in Buffer 2 to the desired concentrations with native detergents [2% n-dodecyl *β*-D-maltoside (Thermo Fischer, Cat. #BN2005) and 0.5% digitonin (Thermo Fischer, Cat. #BN2006)]. Cathode and anode buffers were prepared according to a standard protocol and kept at 4 ^°^C. Prior to loading, NativePAGE™ 0.5% G-250 Sample Additive (Thermo Fischer, Cat. #BN2004) was added. Bands were resolved on a 4 to 12% Novex Tris-Glycine Gel (Invitrogen). Gels were running on ice at 150 V for 1 h and at 250 V for another 2 to 3 h till the front approached the end of the cassette. NativeMark Unstained Protein Standard (Thermo Fischer, Cat. #LC0725) was used as a reference for molecular weight determination. Gels were fixed, stained with Imperial Blue (Thermo Fischer, Cat. #24615) overnight, and washed thoroughly in water until bands were resolvable. Bands were visualized on an Epson Perfection V700 Photo scanner.

## Supplementary Material

Appendix 01 (PDF)

## Data Availability

Python script for the mass photometry data analysis and statistics is available at https://github.com/tobiasjj/iscam_analysis (84). All other data are included in the manuscript and/or *SI Appendix*.
