# Supplementary material for: Monomers and short oligomers of human RAD52 promote single-strand annealing
Source: Proc Natl Acad Sci U S A. 2025 Apr 4;122(14):e2420771122. doi: 10.1073/pnas.2420771122 (PMC12002259; doi:10.1073/pnas.2420771122)
Supplement: Supplementary file 1 — Appendix 01 (PDF) [file pnas.2420771122.sapp.pdf]

Supporting Information for

Monomers and short oligomers of human RAD52 promote single-strand annealing

Kharlamova *et al.*

Corresponding Authors: Philipp Kukura and Erik Schäffer
E-mail: philipp.kukura@chem.ox.ac.uk and erik.schaeffer@uni-tuebingen.de

This PDF file includes:

Supporting text
Figs. S1 to S14
Tables S1 to S5
SI References

Contents

1 **Proteins are functional** **2**
- A At micromolar concentrations, rings and their aggregates form 2
- B RAD52 binds cooperatively to ssDNA 2
- C RAD52 promotes single-strand annealing . . . 2

2 **DNA product yield as a function of DNA length and concentration** **2**

3 **RAD52 monomers and short oligomers bind to ssDNA** **3**

4 **ssDNA excess disassembles rings and aggregates** **3**

Supporting Information Text

1. Proteins are functional

A. At micromolar concentrations, rings and their aggregates form. At micromolar concentrations, blue native gels showed prominent bands that we attribute to rings and their aggregates with the lowest-molecular weight band corresponding to a single ring (Fig. S1). Bands associated with aggregates of rings decreased in intensity with larger aggregate size. Both proteins ran at higher molecular weights than predicted as observed previously (1). Furthermore, RAD52 ring bands were smeared out and more intense compared to the RAD52(209) ones. These observations are consistent with an inhomogenous ring quaternary structure of RAD52 and its C-terminal domain promoting aggregation at micromolar concentrations (2), respectively.

B. RAD52 binds cooperatively to ssDNA. To measure binding affinities of the proteins to ssDNA, we used electrophoretic mobility shift assays (Fig. S2). First, we tested the ability of both proteins to bind to 32 nucleotide (nt)-long ssDNA at 37 °C. At low protein concentration, there were only small shifts of the ssDNA bands indicating that there were either no or weak, transient interactions with the proteins (insets Fig. S2). With increasing protein concentration, ssDNA bound to the proteins and migrated slower. However, shifts did not result in discrete bands but in a smear on the gels indicating transient interactions of various strengths and/or different sizes of associated proteins. We determined the amount of protein-bound ssDNA as a function of concentration (Materials and Methods) and fitted a Hill equation (lines in Fig. S2) to the data. The K_d-values were about 250 nM and 135 nM for RAD52(209) and RAD52, respectively, indicating about a two-fold higher ssDNA binding affinity for RAD52 (see Table S2 for fit parameters). The Hill coefficients were about three (3.1 ± 0.5 and 2.7 ± 0.2 for RAD52(209) and RAD52, respectively). These coefficients are significantly different from 1 (p-value of 0.004 and less than 0.0001, respectively) implying cooperative binding of a few monomers (3). Ring binding would have resulted in a steep rise in the bound fraction with a very high cooperativity, i.e. with the Hill coefficient on the order of 10, which we did not observe, indicating that the rings are not the preferential substrate for protein-DNA interaction (3). As a reference, the dashed and dotted lines in Fig. S2c show how non-cooperative and ring binding curves with the same K_d as the one for RAD52 look like (Hill coefficient of one (Michaelis-Menten) and 11 (rings), respectively). Thus, our ssDNA binding curves are inconsistent with a ssDNA-ring-binding mechanism, but more consistent with monomers and/or short oligomers binding sequentially or independently (3, 4). While Hill-equation fit parameters were similar for longer ssDNA (Fig. S2d), experiments performed at room temperature showed a lower cooperativity (Fig. S2a,b and Table S2). An increase in cooperativity with increasing temperature suggests that ssDNA-RAD52 complex formation requires the transition over an activation barrier. Affinities and cooperativity are expected to depend on the RAD52 oligomeric state and length of the DNA substrate (5, 6). To summarize, both RAD52(209) and RAD52 bind ssDNA cooperatively. Once a monomer or short oligomer is bound, the affinity for further binding is increased. Since allostery is unlikely to be present for non-neighboring binding sites on the oligonucleotide, the cooperativity implies that RAD52 molecules bind next to each other forming a ssDNA-RAD52 complex. Cooperative binding was also observed for RAD52 binding to RPA-coated strands (7, 8).

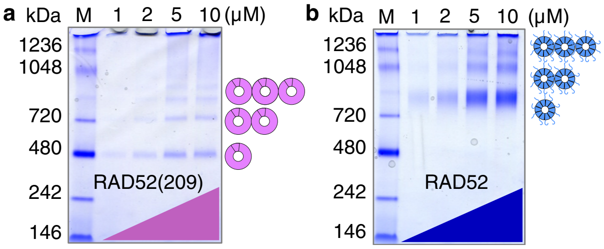

Fig. S1. Blue native gels of **(a)** RAD52(209) and **(b)** RAD52 show bands that we attribute to rings and their aggregates at micromolar concentrations (M: marker).

C. RAD52 promotes single-strand annealing. To determine annealing rates, we incubated complementary strands with the proteins (Fig. S3, see Materials and Methods). With a protein excess (100 nM) over DNA (10 nM) and incubation of only one strand with the proteins before adding the complementary strand, both RAD52(209) and RAD52 promoted annealing. With increasing annealing time, dsDNA bands of electrophoretic mobility shift assays became more intense (Fig. S3a). After quantifying band intensities, we could fit a second-order rate equation to the annealing data consistent with previous observations on the reaction kinetics indicating that collisions of ssDNA-RAD52 complexes with the complementary ssDNA are rate limiting (9) (Fig. S3b). Both RAD52(209) and RAD52 increased the annealing rate compared to the control of hybridization without protein about 6- and 30-fold, respectively (Table S3).

The binding and annealing assays together confirm the functionality of the proteins.

2. DNA product yield as a function of DNA length and concentration

The data in Fig. 2 were obtained with complementary 32-nt and 60-nt long oligonucleotides with 10 nM of each oligonucleotide. We tested how the length and concentration of the oligonucleotides affected the product yield of the annealing reaction normalized to the hybridization reaction without pro-

tein (Fig. S4). Experiments were done in the same manner as in Fig. 2. We found that both RAD52 and RAD52(209) promoted annealing in a similar manner for all tested pairs. RAD52(209) had the highest product yield at 200 nM independent of the oligonucleotide length (Fig. S4a). Compared to the (32+60)-pair used in Fig. 2, shorter and longer oligonucleotides did not promote annealing at 50 nM. Otherwise trends were similar. RAD52 had the highest product yield at 100–200 nM with the maximum covering a wider concentration range and a better annealing performance than RAD52(209) at lower concentrations. Why the (12+26)-pair had a very high yield at 200 nM is unclear. Overall, there was not a strong dependence of the normalized annealing product yield on oligonucleotide length for the tested pairs.

We next tested how product yield depended on the oligonucleotide concentration for the (48+60)-pair (Fig. S4b). For RAD52(209), the highest yields resulted from using a 5 nM oligonucleotide concentration. For 5 nM and 10 nM, the maximum yield was at 200 nM RAD52(209) or about 18 nM ring concentration. Thus, the maximum was at a ring-to-ssDNA ratio of 3.6 and 1.8, respectively. For 25 nM, the maximum shifted to 500 nM (45 nM rings) corresponding to a ring-to-ssDNA ratio of 1.8. For RAD52, the highest yields also resulted from using a 5 nM oligonucleotide concentration. For all concentrations, the maximum was at ≈100 nM RAD52 with the maximum being wider than for RAD52(209). Assuming on average 10 protomers per RAD52 ring, the ring-to-ssDNA ratio was 2, 1, and 0.5 for the three tested concentrations, respectively. Overall, there was not a consistent ratio that led to optimal annealing.

In summary, the length of the used oligonucleotides had little effect on the annealing activity of both proteins. Lower oligonucleotide concentrations led to higher normalized product yields. However, increasing the oligonucleotide concentration also increased the intrinsic hybridization rate without protein. Thus, the normalization procedure reduced the relative product yield for higher oligonucleotide concentrations. Increased hybridization rates limited the maximum oligonucleotide concentration we could test.

3. RAD52 monomers and short oligomers bind to ssDNA

To enhance binding of ssDNA-bound RAD52 complexes, we used a modified, positively charged glass surface for mass photometry (10). As expected, on these surfaces, ssDNA-RAD52 complexes bound rigidly. We could detect a shift in molecular weight not only for rings but also for monomers and short oligomers (Fig. S11). We did not use these surfaces for the other measurements because RAD52 without any bound DNA mostly diffused on these surface preventing mass determination. Thus, also a control of the protein without DNA on these surfaces was not reliable. The first peak in the mass histogram of Fig. S11a is consistent with the molecular weight of a RAD52 monomer without ssDNA. The second peak, we attribute to a monomer bound to ssDNA. This peak's molecular weight is significantly different from the sequence molecular weight of a dimer without DNA (dashed vertical line marked with a two). For subsequent peaks, we assigned shifts in consecutive order to RAD52 oligomers. In this manner, the shifted decamer peak still had the highest abundance comparable to the data without ssDNA (Fig. 4). Shifts were consistent with two single strands bound per monomer or short oligomer up to heptamers and an increasing number of strands—up to seven ssDNA—bound to single rings (Fig. S11b). More than two bound strands suggests that some complexes have DNA strands that do not fully wrap around rings, but have parts dangling freely from the rings. Such DNA tethers may increase the negative charge of the complexes and prevent their binding to normal, negatively charged glass surfaces for which we detected only rings with two bound ssDNA for RAD52(209). Together this data shows that also RAD52 monomers and short oligomers can bind ssDNA.

4. ssDNA excess disassembles rings and aggregates

To test whether more ssDNA could disassemble rings as previously suggested (12), we increased the ratio of ssDNA to protein (Fig. S13). With 100 nM of protein and adding increasing amounts of ssDNA (10 nM, 100 nM or 200 nM), the molecular-weight shift of the detected ring peak did not significantly change with more ssDNA present. On average, about two oligonucleotides were bound. We did not observe a shift for monomers and short oligomers but a more than 10-fold increase in their counts at the highest used ssDNA concentration (bottom row in Fig. S13). Also, the counts of larger species and aggregates decreased; and, for RAD52(209), the number of rings increased. We attribute the increase in ring count to a disassembly of larger species and aggregates. The increase in the number of monomers short oligomers suggests that rings were disassembled with an excess of ssDNA consistent with previous work (12).

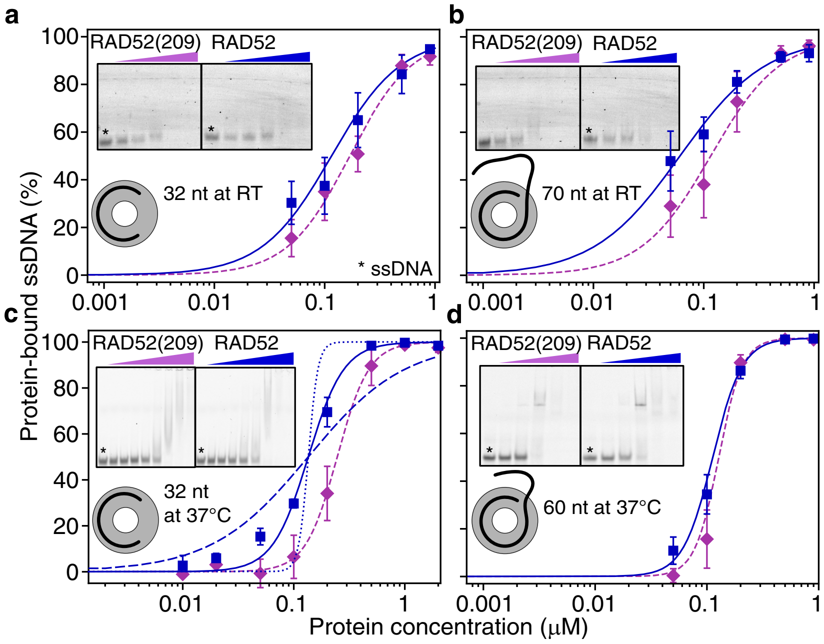

Fig. S2. Electrophoretic mobility shift assays of 10 nM ssDNA binding to RAD52(209) and RAD52. Three different lengths were tested, 32, 60, and 70 nt (see Table S1 for sequences). DNA binding was tested at 25 °C with **(a)** 32 nt (5 repeats) and **(b)** 70 nt (4 repeats), and at 37 °C with **(c)** 32 nt (4 repeats) and **(d)** 60 nt (3 repeats). Hill equations (RAD52: solid lines, RAD52(209): short dashed lines) were fitted to the values (mean ± SEM, see Table S2 for fit parameters). Blue long dashed and dotted lines in **(c)** are plots of the Hill equation with a fixed K_d and Hill coefficient of 1 and 11, respectively. Exemplary binding gels are shown as insets with the first column being a control (no protein, marked with a star) and the others correspond to the plotted concentrations.

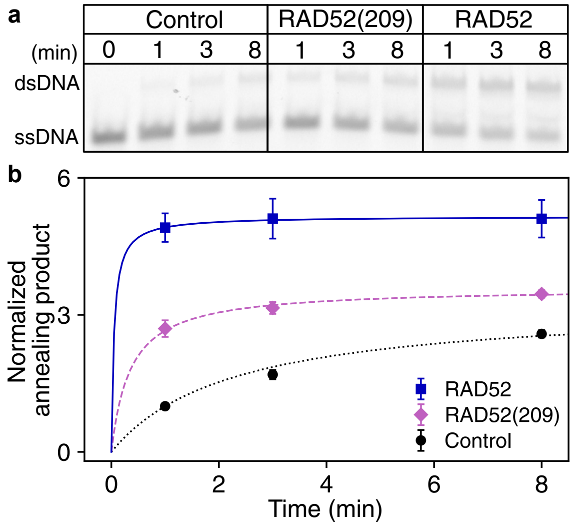

Fig. S3. RAD52 annealing assays. **(a)** Exemplary annealing gel using complementary 32-nt long, 3'-ATTO680-labeled and 60-nt long, unlabeled oligonucleotides for the annealing reaction as a function of time (Control: no protein). Protein and DNA concentrations were 100 nM and 10 nM, respectively. The reaction was the same as in Fig. 2c but terminated at earlier time points (see Methods: Terminated DNA annealing assays). **(b)** Annealing product as a function of time (mean ± SEM, 3 repeats). Lines are fits of second-order reaction kinetics (Table S3). Data are normalized to the control at 1 min.

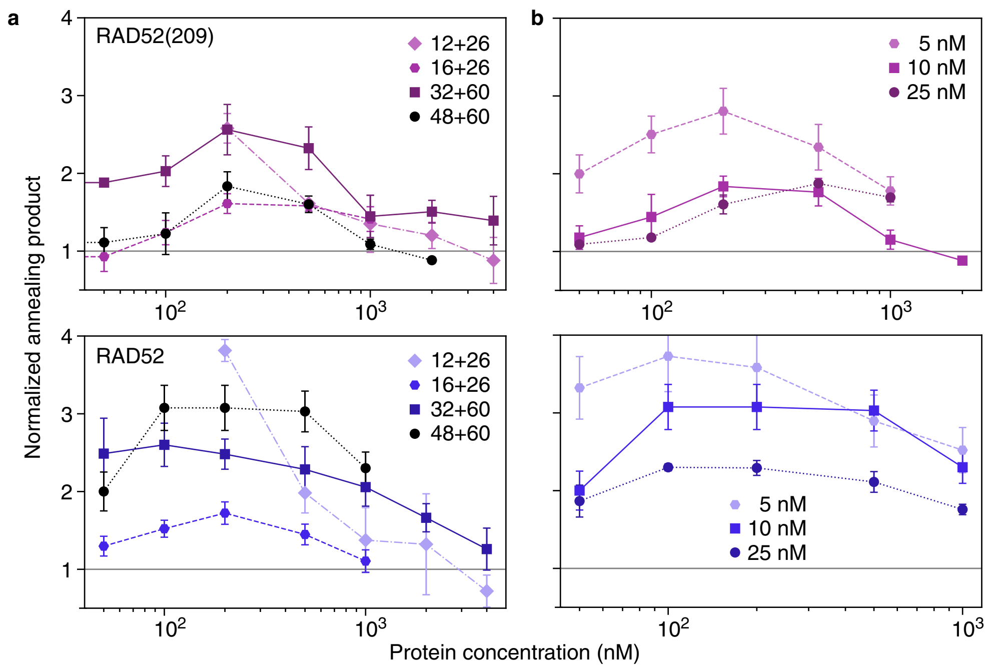

Fig. S4. DNA product yield as a function of DNA length and concentration. Annealing product of RAD52(209) (top row) and RAD52 (bottom row) for different oligonucleotide lengths indicated in the legend in nucleotides **(a)** and concentrations of both strands for the (48 + 60)-pair **(b)**. Proteins were preincubated with the longer oligonucleotide and the complementary strand added afterwards as in Fig. 2c. Intensities of dsDNA bands were normalized to the control reaction (intrinsic annealing without protein) and averaged. All data points are mean values ± SEM (at least 3 repeats).

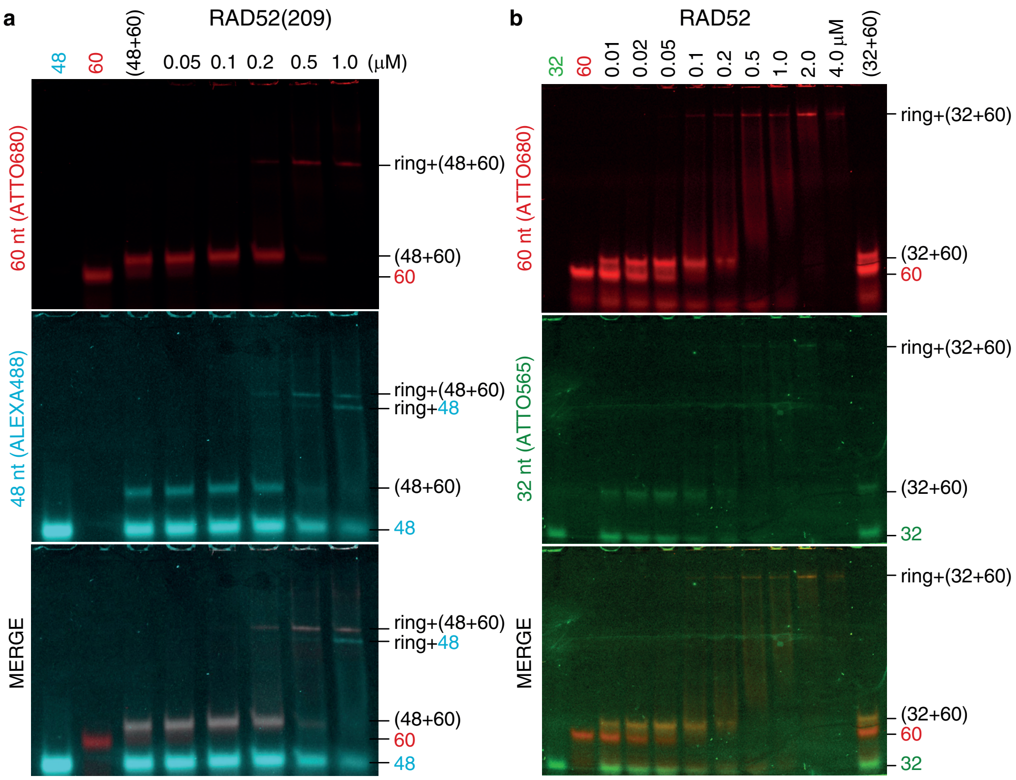

Fig. S5. Annealing gels of fluorescently-labeled oligonucleotides. Native gels of complementary oligonucleotides with increasing concentrations of **(a)** RAD52(209) and **(b)** RAD52, respectively (the 60 nt-long strand was incubated with the protein before adding the other strand). The control columns labeled with the oligonucleotide lengths were without protein.

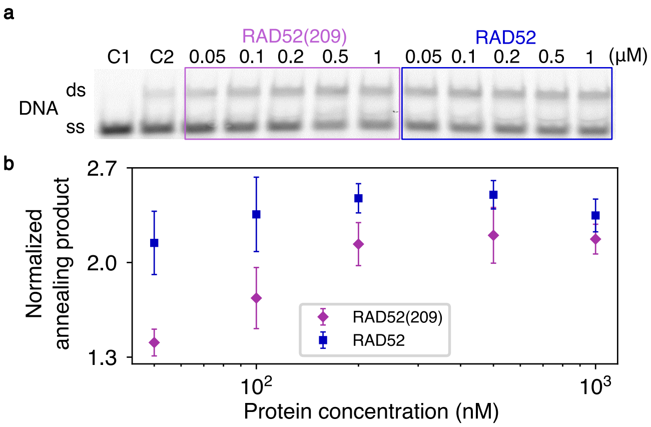

Fig. S6. dsDNA product yield of the annealing reaction in the mass photometry buffer. Experiments were identical to the ones in Fig. 2c using 32- and 60-nt long oligonucleotides except for the different buffer (photometry buffer: 20 mM Tris-HCl, 50 mM NaCl, pH 7.5 vs. buffer for DNA binding and annealing assays (as in Fig. 2): 25 mM Tris-Acetate, 2 mM $Mg(OAc)_2$, 1 mM DTT, pH 7.5, see Materials and Methods). **(a)** Annealing gel of electrophoretic mobility shift assay. The 60-nt strand was incubated with the protein before mixing it with the other strand. Control C1: labeled oligonucleotide. C2: annealing control used for normalization, i.e. hybridizing reaction of both oligonucleotides without protein. **(b)** Quantification of the band intensities attributed to the dsDNA normalized by the Control C2. Values are comparable to the data in Fig. 2 suggesting that annealing did not depend on the composition of the two buffers. All data points are mean values $\pm$ SEM (3 repeats) and oligonucleotide concentrations of both strands were 10 nM.

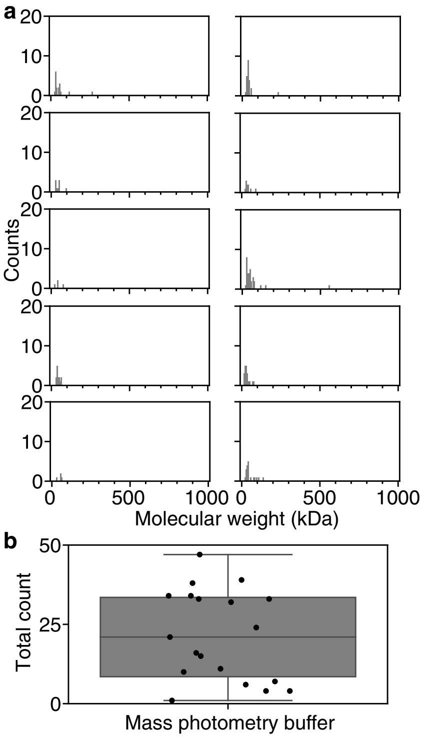

Fig. S7. Buffer control. **(a)** Mass histograms of the mass photometry buffer alone acquired on different days. Each histogram represents one reading. **(b)** Total number of counts from mass the histograms. Each reading is a black circle (mean $\pm$ SEM).

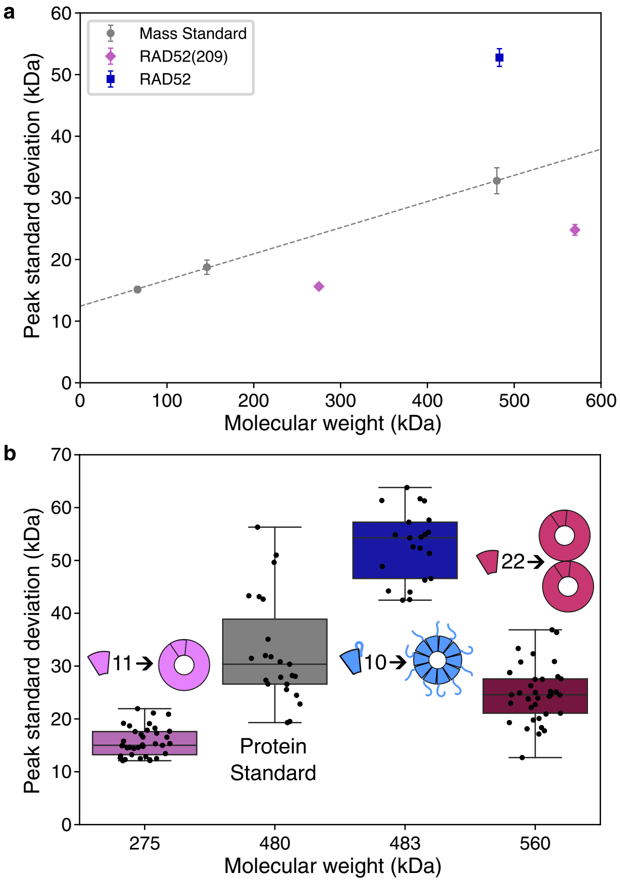

Fig. S8. Standard deviation of mass photometry peaks. **(a)** Standard deviation of calibration proteins (gray circles, mean $\pm$ SEM) increased linearly with molecular weight of the proteins (dashed line with a slope of 0.042 kDa^{-1} and an intercept of 12.43 kDa). The peaks of the RAD52(209) undecamer and the cluster of two undecamers had a significantly smaller standard deviation (magenta diamonds) compared to the calibration standards. The standard deviation of the RAD52 ring peak when fitted with a *single* Gaussian of 53 ± 2 kDa (mean $\pm$ SEM, 21 readings measured on 7 days) was significantly larger compared to the calibration standard of 33 ± 2 kDa (23 readings on 11 days) and the RAD52(209) undecamer peak of 15.6 ± 0.5 kDa (37 readings on 12 days). The most plausible explanation for the large standard deviation is that the RAD52 ring peak is a convolution of multiple peaks of rings containing a different number of protomers having smaller standard deviations comparable to the calibration standards. **(b)** Box-and-whisker plot and distribution of standard deviation of mass photometry peaks from the individual measurements.

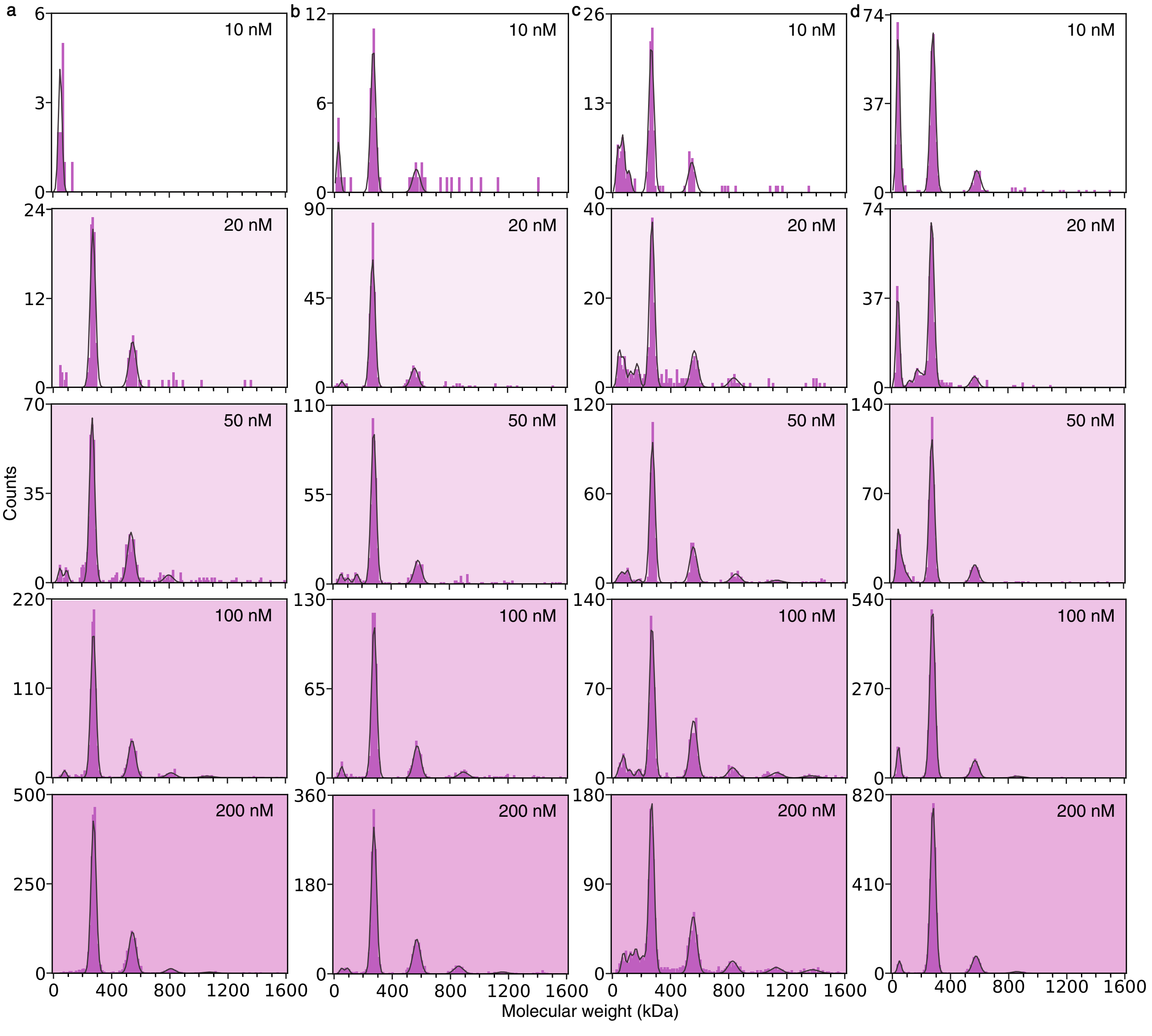

Fig. S9. Oligomeric states of RAD52(209) at different concentrations indicated in the plots on different days **(a)**–**(d)**. Each plot is a cumulative distribution from 2–3 measurements from the same day.

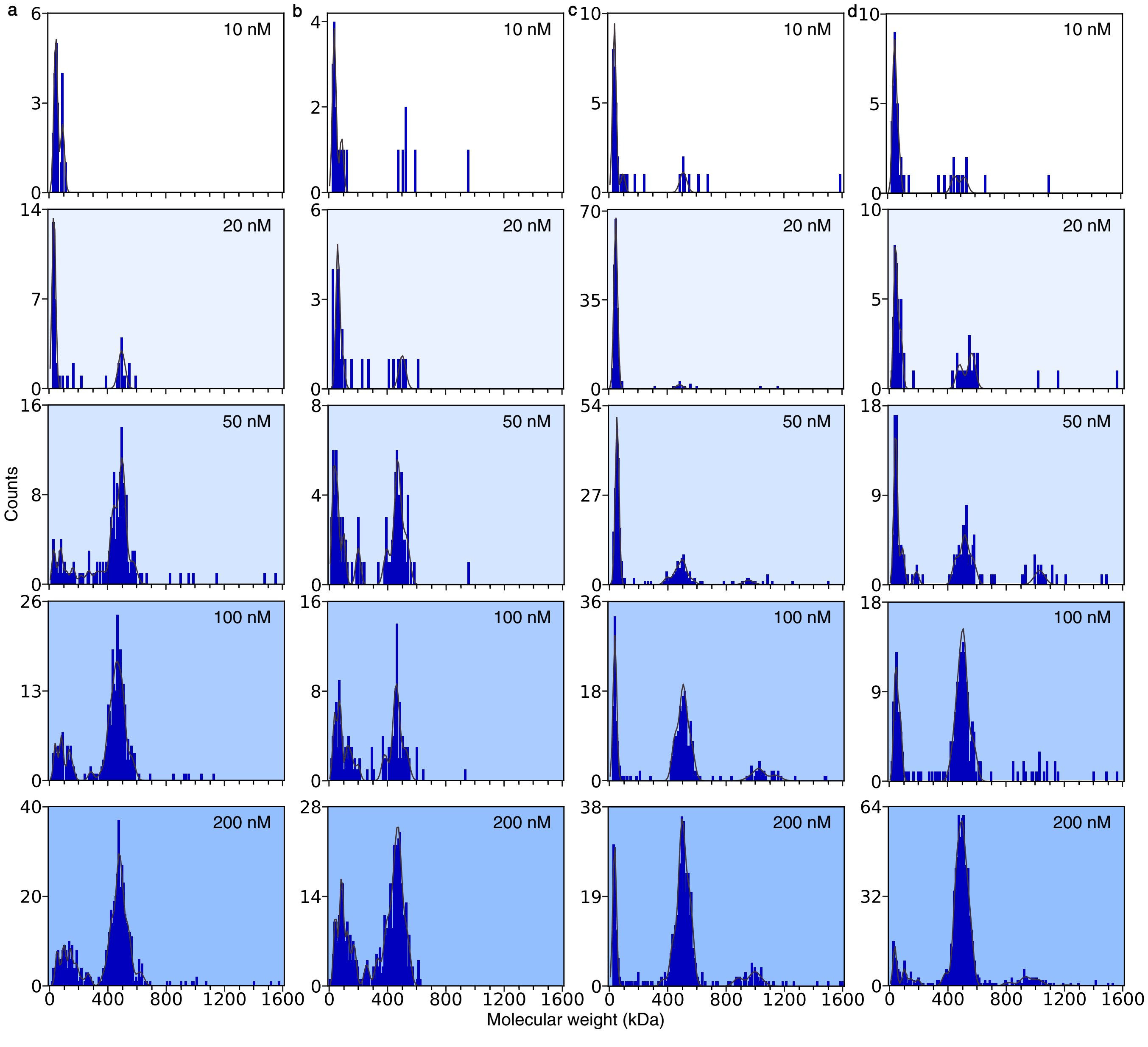

Fig. S10. Oligomeric states of RAD52 at different concentrations indicated in the plots on different days **(a)**–**(d)**. Each plot is a cumulative distribution from 2–3 measurements from the same day.

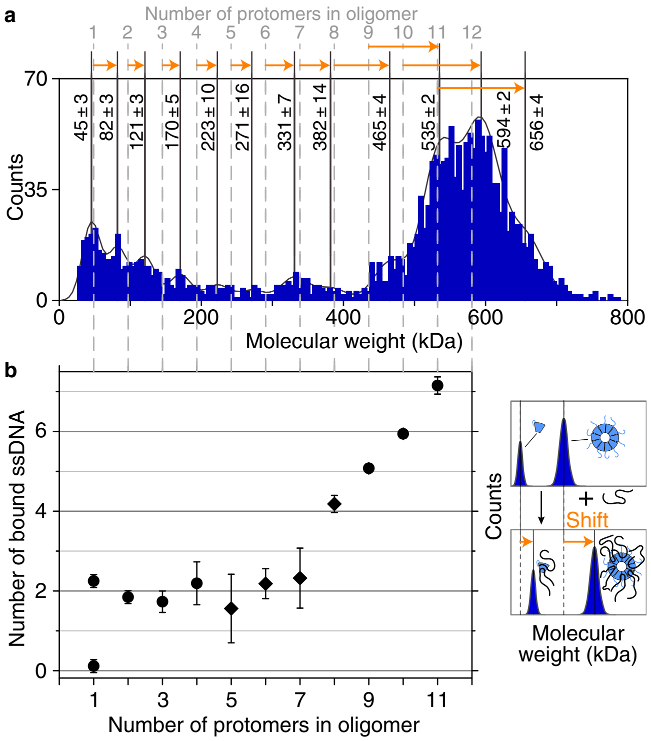

Fig. S11. DNA binding with mass photometry on positively charged surfaces. **(a)** Mass histogram of RAD52 incubated with ssDNA on an APTES-coated surface (10) (100 nM protein and 100 nM unlabeled 60 nt-long, 18.6 kDa ssDNA). Dashed vertical lines correspond to the sequence-based mass of RAD52 oligomers and solid lines to detected peaks with marked molecular weights (centers ± SEM). **(b)** Molecular weight shift of peaks (orange arrows in (a) illustrated to the right) relative to measurements (circles) or the sequence-based mass (diamonds) in the absence of ssDNA (Table S4) normalized to the molecular weight and different mass sensitivity (10) of ssDNA (a 7 % correction). Error bars correspond to SEMs of (a).

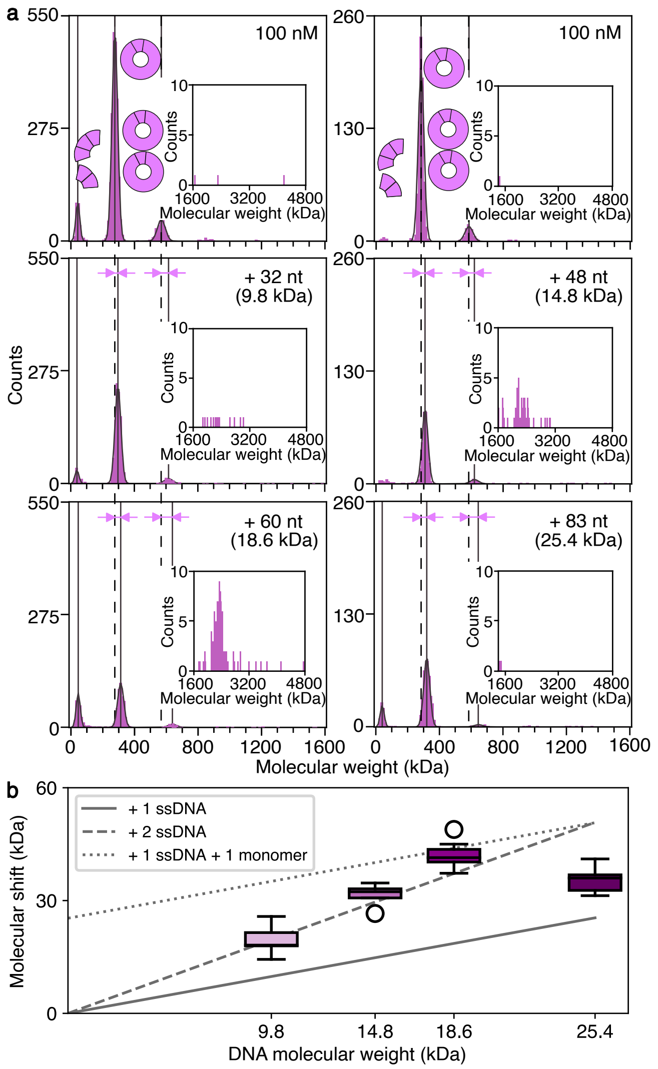

Fig. S12. Mass photometry shifts of ssDNA binding to RAD52(209) rings. **(a)** RAD52(209) (100 nM) incubated with ssDNA (10 nM) of different lengths indicated in the panels. **(b)** Corresponding molecular weight shifts of single rings marked with pink arrows in (b) (mean ± SEM). Lines indicate different expected scaling behavior.

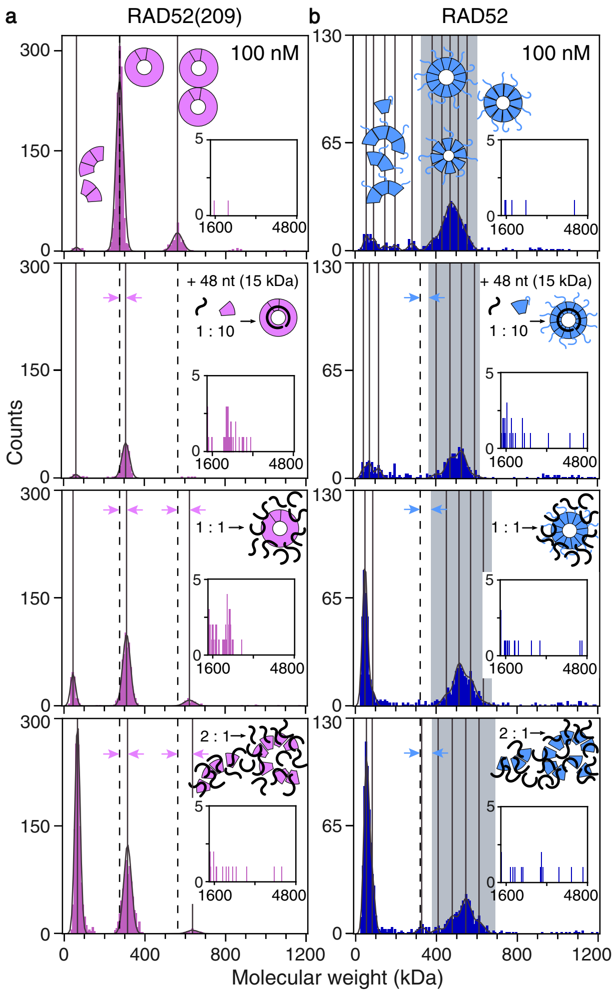

Fig. S13. Mass photometry with an excess of DNA over protein. Mass histograms of **(a)** RAD52(209) and **(b)** RAD52 (both 100 nM) before (top row) and after the addition of ssDNA (unlabeled 48 nt; 10 nM, 100 nM, and 200 nM, respective following rows). Different protein-to-DNA ratios are illustrated as insets. Vertical solid lines indicate peak centers. Dashed lines mark RAD52(209) peak centers or the start of the RAD52 ring peak without added DNA. Molecular weight shifts upon DNA binding correspond to distance between arrowheads. Each dataset is a cumulative distribution of three measurements.

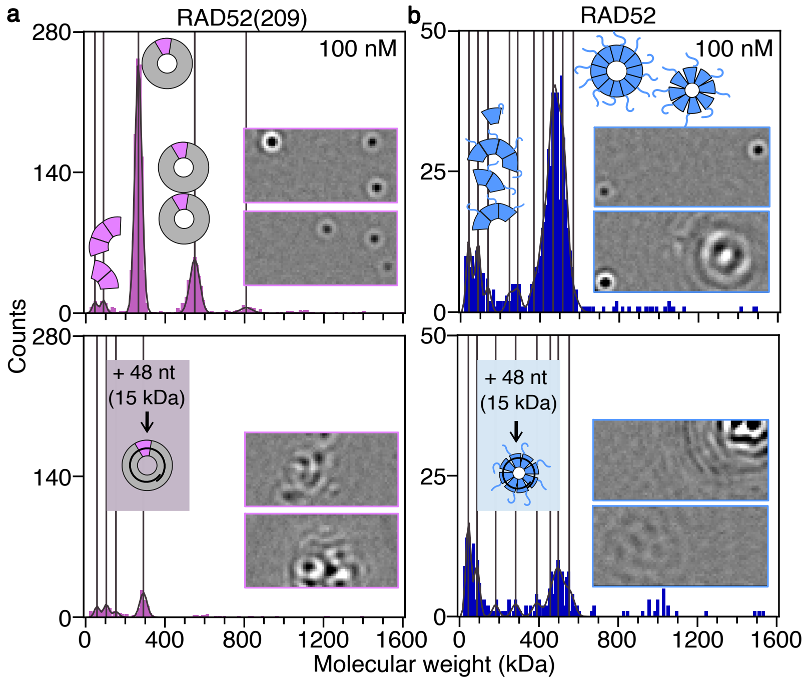

Fig. S14. Mass photometry data of **(a)** RAD52(209) and **(b)** RAD52 at 100 nM (top row) and with ssDNA bound (48 nt, 10 nM, bottom row). Formation of large clusters was observed (insets). Each dataset is a cumulative distribution from three repeats.

Table S1. List of oligonucleotides.

Name	Sequence
32 nt 3'-ATTO565	GCTCTAAGCCATCCGCAAAAATGACCTCTTAT
48 nt 3'-ALEXA488	GCAATTAAGCTCTAAGCCATCCGCAAAAATGACCTCTTATCAAAAGGA
60 nt 3'-ATTO680	TCCTTTTGATAAGAGGTCATTTTTGCGGATGGCTTAGAGCTTAATTGCTTTTTTTTTTTT
70 nt 3'-ATTO565	TTTAATAATTTACTTTATTTTCTATGTCTATTCATTTACTTATTGTGTATTATCCTTATACTTACTTAC
83 nt[a]	TTTATATAATTTACTTTATTTTCTATGTCTATTCATTTACTTATTGTGTATTATCCTTATACTTACTTAC TTTATGTTTATTT

[a] Mazina *et al.* (13)

Table S2. Hill equation fit parameters for the single-stranded DNA binding reactions (Fig. S2). For RAD52 binding to 70 nt at 25 °C, a fit to the Michaelis-Menten equation ($H = 1$) was best. Fits were weighted with the SEM. V_{max} was fixed to 100 %.

	RAD52(209)		RAD52	
Number nt (T in °C)	K_d	H	K_d	H
32 nt (25)	169 ± 24	1.5 ± 0.2	115 ± 22	1.4 ± 0.2
70 nt (25)	109 ± 23	1.6 ± 0.3	53 ± 8	-
32 nt (37)	246 ± 33	3.1 ± 0.5	135 ± 4	2.7 ± 0.2
60 nt (37)	127 ± 10	4.1 ± 0.4	112.4 ± 0.1	3.3 ± 0.2

Table S3. Fit parameters for second-order reaction kinetics $2Akt/(1/A + 2kt)$ in Fig. S3

Reaction	Amplitude A	Rate constant k (min^{-1})
Control	3.3 ± 0.2	0.068 ± 0.009
RAD52(209)	3.59 ± 0.06	0.39 ± 0.08
RAD52	5.16 ± 0.03	2.0 ± 0.3

Table S4. Molecular weights of RAD52 oligomers. The table contains estimated molecular weights based on the protein sequence (M_w^{sequence}) and the values obtained by mass photometry (M_w^{measured}, mean ± SEM). To measure the molecular weight of RAD52 oligomers, we used data from 4 days. On each day, the protein was measured at 5 concentrations (10, 20, 50, 100, and 200 nM). Fitted molecular weights of oligomers with the same stoichiometry were grouped together and averaged (N: number of measurements). The reasonable agreement between the measured and expected molecular weights supports the robustness of our fitting procedure.

Oligomer	M_w^{sequence} (kDa)	M_w^{measured} (kDa)	N
Monomer (1)	48.4	43 ± 2	20
Dimer (2)	96.8	89 ± 3	20
Trimer (3)	145.2	140 ± 5	6
Tetramer (4)	193.6	185 ± 4	6
Nonamer (9)	435.6	447 ± 3	11
Decamer (10)	484	491 ± 2	16
Undecamer (11)	532.4	532 ± 2	10
Dodecamer (12)	580.9	573 ± 5	8

Table S5. Shifts of molecular weights of RAD52(209) single ring peak after binding of ssDNA of different lengths and during the annealing reaction (N: number of repeats). The protein and ssDNA concentrations were 100 nM and 10 nM, respectively.

Length in nt (kDa)	MW shift (kDa, mean ± SEM)	N
32 (9.8)	20 ± 1	7
48 (14.8)	32 ± 2	6
60 (18.6)	42 ± 1	6
83 (25.4)	40 ± 4	6
60 (preincubated) + 32 (28.4)	42 ± 2	9

References

1. M Honda, Y Okuno, J Yoo, T Ha, M Spies, Tyrosine phosphorylation enhances RAD52-mediated annealing by modulating its DNA binding. *EMBO J.* **30**, 3368–3382 (2011).
2. W Ranatunga, et al., Human RAD52 exhibits two modes of self-association. *J. Biol. Chem.* **276**, 15876–15880 (2001).
3. JN Weiss, The Hill equation revisited: uses and misuses. *FASEB J.* **11**, 835–841 (1997).
4. MI Stefan, N Le Novère, Cooperative binding. *PLoS Comput. Biol.* **9**, e1003106 (2013).
5. IR Epstein, Cooperative and non-cooperative binding of large ligands to a finite one-dimensional lattice: A model for ligand-ougonucleotide interactions. *Biophys. Chem.* **8**, 327–339 (1978).
6. M Ander, S Subramaniam, K Fahmy, A Francis Stewart, E Schäffer, A single-strand annealing protein clamps DNA to detect and secure homology. *PLoS Biol.* **13**, 1–23 (2015).
7. B Gibb, et al., Protein dynamics during presynaptic-complex assembly on individual single-stranded DNA molecules. *Nat. Struct. Mol. Biol.* **21**, 893–900 (2014).
8. JC Bell, SC Kowalczykowski, Mechanics and single-molecule interrogation of DNA recombination. *Annu. Rev. Biochem.* **85**, 193–226 (2016).
9. UH Mortensen, C Bendixen, I Sunjevaric, R Rothstein, DNA strand annealing is promoted by the yeast Rad52 protein. *Proc. Natl. Acad. Sci. U. S. A.* **93**, 10729–10734 (1996).
10. Y Li, WB Struwe, P Kukura, Single molecule mass photometry of nucleic acids. *Nucleic Acids Res.* **48**, e97–e97 (2020).
11. X Deng, et al., Human replication protein A-Rad52-single-stranded DNA complex: Stoichiometry and evidence for strand transfer regulation by phosphorylation. *Biochemistry* **48**, 6633–6643 (2009).
12. OM Mazina, H Keskin, K Hanamshet, F Storici, AV Mazin, Rad52 inverse strand exchange drives RNA-templated DNA double-strand break repair. *Mol. Cell* **67**, 19–29.e3 (2017).
